# Supplementary material for: Engineering Tumor Stroma Morphogenesis Using Dynamic Cell-Matrix Spheroid Assembly
Source: bioRxiv. 2024 Mar 22:2024.03.19.585805. Preprint. [Version 1] doi: 10.1101/2024.03.19.585805 (PMC11188064; doi:10.1101/2024.03.19.585805)
Supplement: Supplement 1 [file media-1.pdf]

Supplementary Materials

Engineering Tumor Stroma Morphogenesis Using Dynamic Cell-Matrix Spheroid Assembly

Michael J. Buckenmeyer[1†], Elizabeth A. Brooks[1†], Madison S. Taylor[1], Liping Yang[2], Ronald J. Holewinski[3], Thomas J. Meyer[4], Mélissa Galloux[5], Marcial Garmendia-Cedillos[6], Thomas J. Pohida[6], Thorkell Andresson[3], Brad St. Croix[2], Matthew T. Wolf[1*]

1 Cancer Biomaterials Engineering Laboratory, Cancer Innovation Laboratory, Center for Cancer Research, National Cancer Institute, National Institutes of Health, Frederick, MD, 21702, USA.
2 Tumor Angiogenesis Unit, Mouse Cancer Genetics Program, National Cancer Institute, National Institutes of Health, Frederick, MD, 21702, USA.
3 Protein Characterization Laboratory, Cancer Research Technology Program, Frederick National Laboratory for Cancer Research, Frederick, MD, 21701, USA.
4 CCR Collaborative Bioinformatics Resource, Center for Cancer Research, National Cancer Institute, National Institutes of Health, Bethesda, MD, 20892, USA.
5 Independent Bioinformatician, Marseille, Provence-Alpes-Côte d'Azur, France
6 Instrumentation Development and Engineering Application Solutions, National Institute of Biomedical Imaging and Bioengineering, National Institutes of Health, Bethesda, MD, 20892, USA.

[†] Authors contributed equally
*Correspondence to: matthew.wolf@nih.gov

Keywords: 3D in vitro modeling, tumor microenvironment, extracellular matrix, biomaterials, spheroids, decellularization

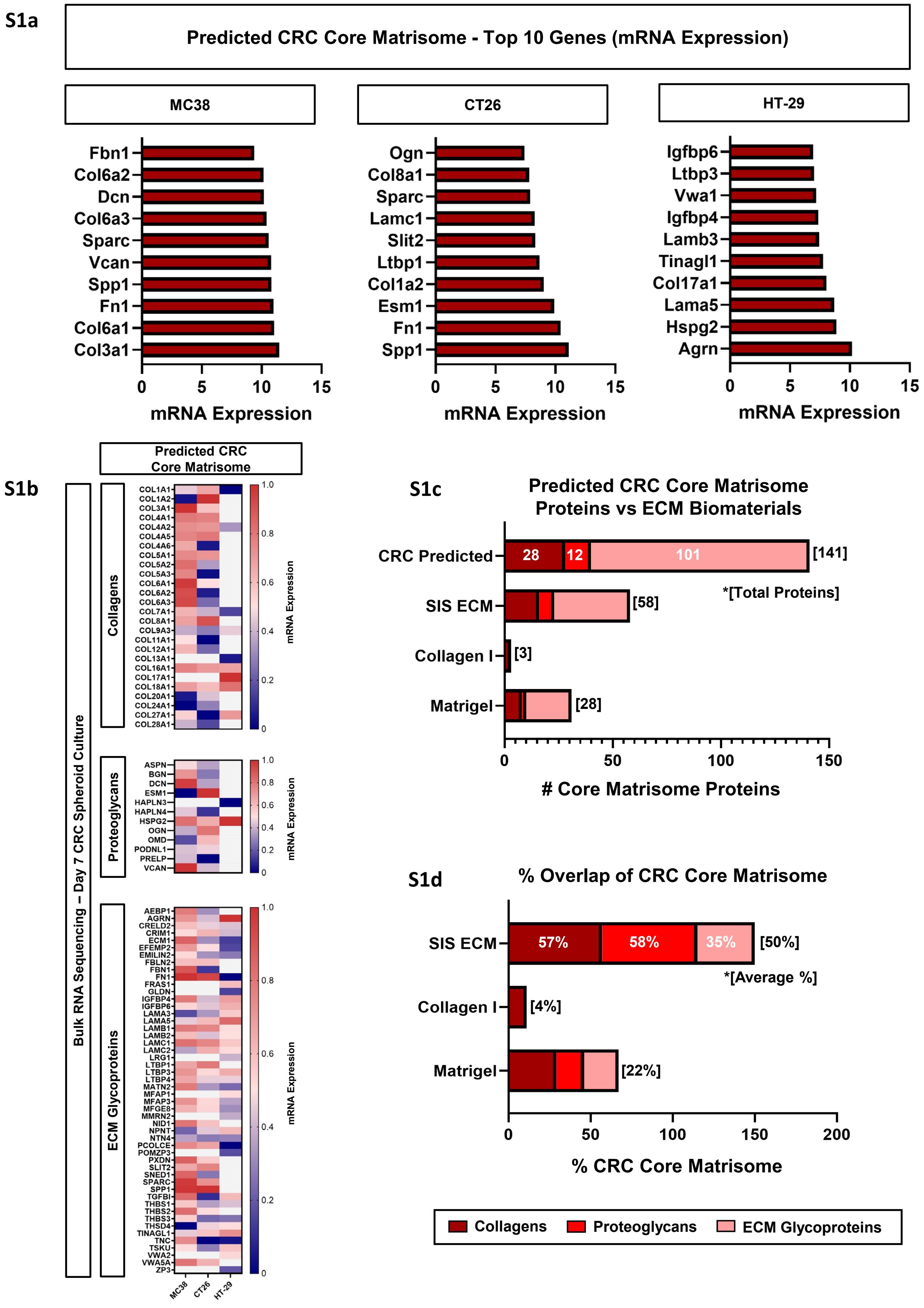
S1a
Predicted CRC Core Matrisome - Top 10 Genes (mRNA Expression)
MC38
Fbn1
Col6a2
Dcn
Col6a3
Sparc
Vcan
Spp1
Fn1
Col6a1
Col3a1
0 5 10 15
mRNA Expression
CT26
Ogn
Col8a1
Sparc
Lamc1
Slit2
Ltbp1
Col1a2
Esm1
Fn1
Spp1
0 5 10 15
mRNA Expression
HT-29
Igfbp6
Ltbp3
Vwa1
Igfbp4
Lamb3
Tinagl1
Col17a1
Lama5
Hspg2
Agrn
0 5 10 15
mRNA Expression
S1b
Predicted CRC Core Matrisome
Bulk RNA Sequencing – Day 7 CRC Spheroid Culture
Collagens
COL1A1 COL1A2 COL3A1 COL4A1 COL4A2 COL4A5 COL4A6 COL5A1 COL5A2 COL5A3 COL6A1 COL6A2 COL6A3 COL7A1 COL8A1 COL9A3 COL11A1 COL12A1 COL13A1 COL16A1 COL17A1 COL18A1 COL20A1 COL24A1 COL27A1 COL28A1
Proteoglycans
ASPN BGN DCN ESM1 HAPLN3 HAPLN4 HSPG2 OGN OMD PODNL1 PRELP VCAN
ECM Glycoproteins
AEBP1 AGRN CRELD2 CRIM1 ECM1 EFEMP2 EMILIN2 FBLN2 FBN1 FN1 FRAS1 GLDN IGFBP4 IGFBP6 LAMA3 LAMA5 LAMB1 LAMB2 LAMC1 LAMC2 LRG1 LTBP1 LTBP3 LTBP4 MATN2 MFAP1 MFAP3 MFGE8 MMRN2 NID1 NPNT NTN4 PCOLCE POMZP3 PXDN SLIT2 SNED1 SPARC SPP1 TGFBI THBS1 THBS2 THBS3 THSD4 TINAGL1 TNC TSKU VWA2 VWA5A ZP3
MC38 CT26 HT-29
mRNA Expression
0 0.2 0.4 0.6 0.8 1.0
S1c
Predicted CRC Core Matrisome Proteins vs ECM Biomaterials
CRC Predicted
28 12 101
[141]
SIS ECM
[58]
*[Total Proteins]
Collagen I
[3]
Matrigel
[28]
0 50 100 150
Core Matrisome Proteins
S1d
% Overlap of CRC Core Matrisome
SIS ECM
57% 58% 35%
[50%]
*[Average %]
Collagen I
[4%]
Matrigel
[22%]
0 50 100 150 200
% CRC Core Matrisome
Collagens
Proteoglycans
ECM Glycoproteins

S1e

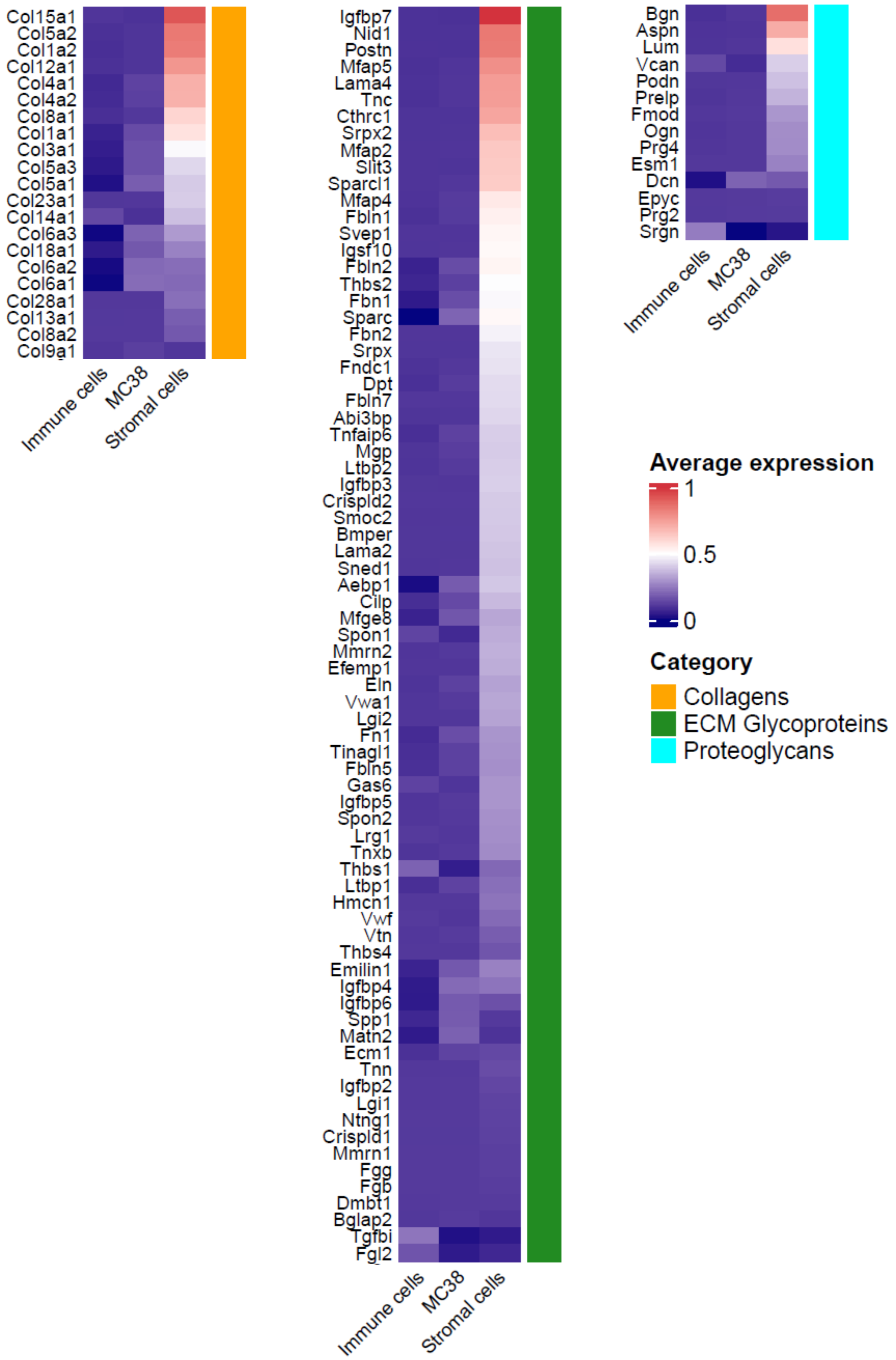

Col15a1
Col5a2
Col1a2
Col12a1
Col4a1
Col4a2
Col8a1
Col1a1
Col3a1
Col5a3
Col5a1
Col23a1
Col14a1
Col6a3
Col18a1
Col6a2
Col6a1
Col28a1
Col13a1
Col8a2
Col9a1
Immune cells
MC38
Stromal cells
Igfbp7
Nid1
Postn
Mfap5
Lama4
Tnc
Cthrc1
Srpx2
Mfap2
Slit3
Sparcl1
Mfap4
Fbln1
Svep1
Igsf10
Fbln2
Thbs2
Fbn1
Sparc
Fbn2
Srpx
Fndc1
Dpt
Fbln7
Abi3bp
Tnfaip6
Mgp
Ltbp2
Igfbp3
Crispld2
Smoc2
Bmper
Lama2
Sned1
Aebp1
Cilp
Mfge8
Spon1
Mmrn2
Efemp1
Eln
Vwa1
Lgi2
Fn1
Tinagl1
Fbln5
Gas6
Igfbp5
Spon2
Lrg1
Tnxb
Thbs1
Ltbp1
Hmcn1
Vwf
Vtn
Thbs4
Emilin1
Igfbp4
Igfbp6
Spp1
Matn2
Ecm1
Tnn
Igfbp2
Lgi1
Ntng1
Crispld1
Mmrn1
Fgg
Fgb
Dmbt1
Bglap2
Tgfbi
Fgl2
Immune cells
MC38
Stromal cells
Bgn
Aspn
Lum
Vcan
Podn
Prelp
Fmod
Ogn
Prg4
Esm1
Dcn
Epyc
Prg2
Srgn
Immune cells
MC38
Stromal cells
Average expression
1
0.5
0
Category
Collagens
ECM Glycoproteins
Proteoglycans

S1f

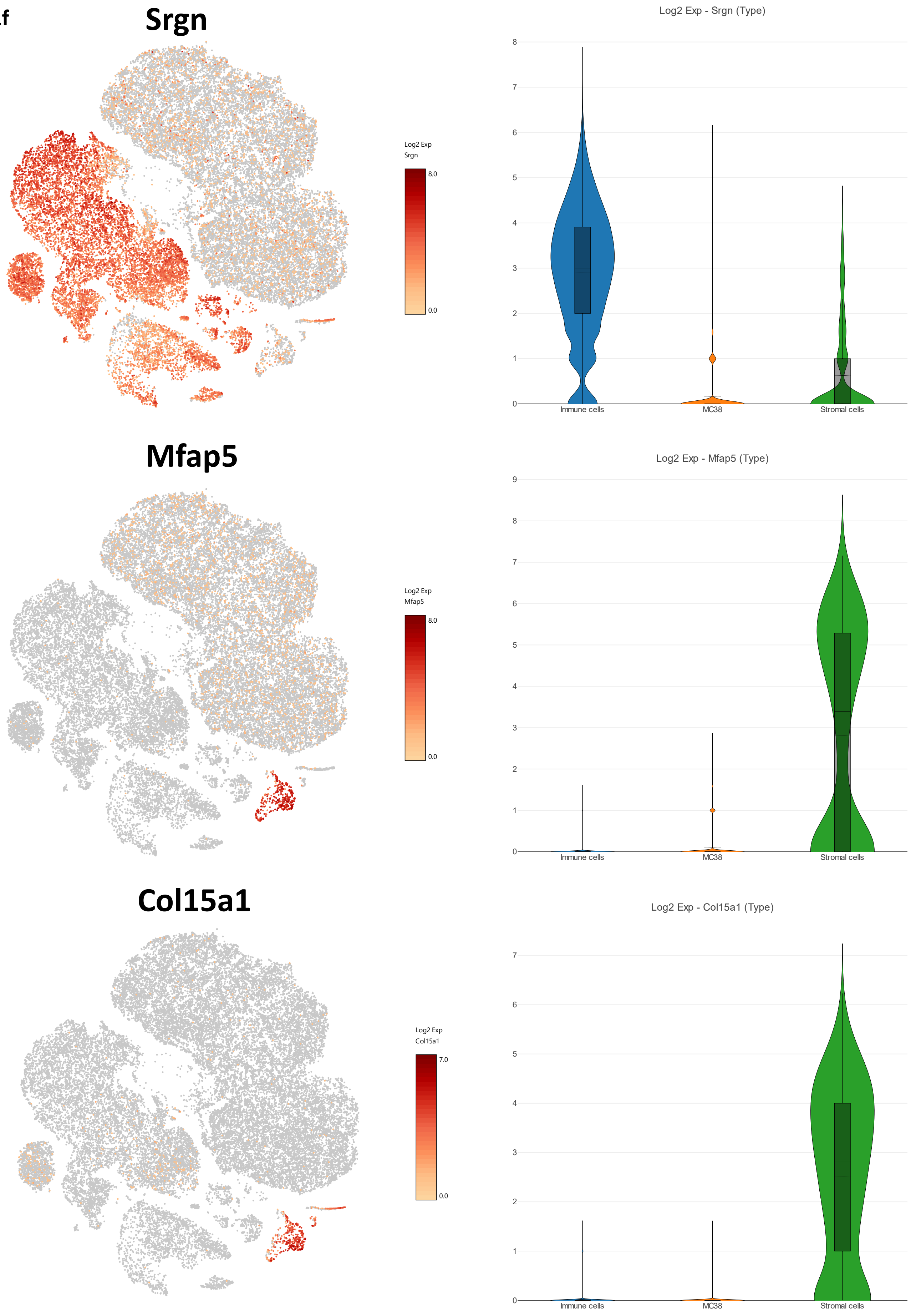
Srgn
Log2 Exp
Srgn
8.0
0.0
Log2 Exp - Srgn (Type)
Immune cells
MC38
Stromal cells
Mfap5
Log2 Exp
Mfap5
8.0
0.0
Log2 Exp - Mfap5 (Type)
Immune cells
MC38
Stromal cells
Col15a1
Log2 Exp
Col15a1
7.0
0.0
Log2 Exp - Col15a1 (Type)
Immune cells
MC38
Stromal cells

S1f (cont.)

Fgl2

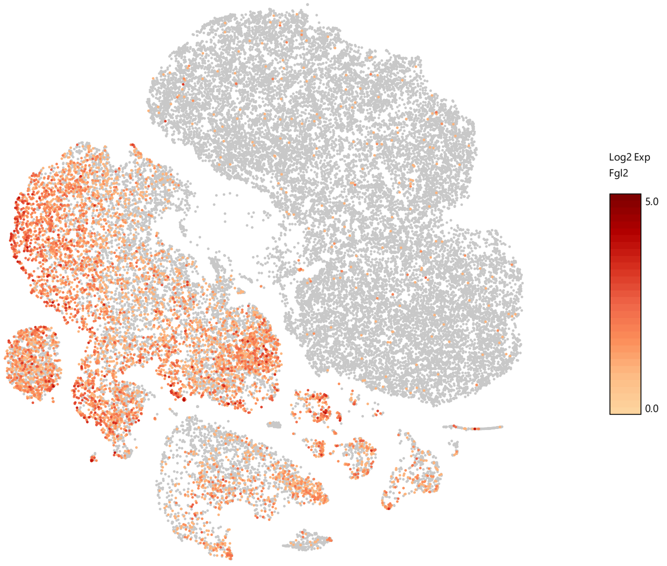

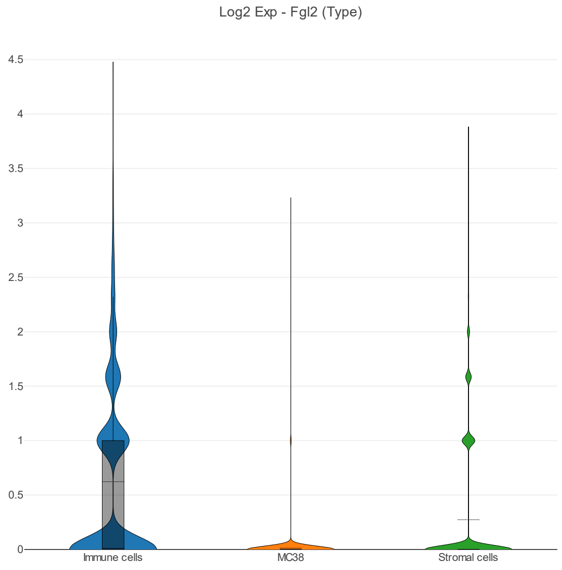

Spon1

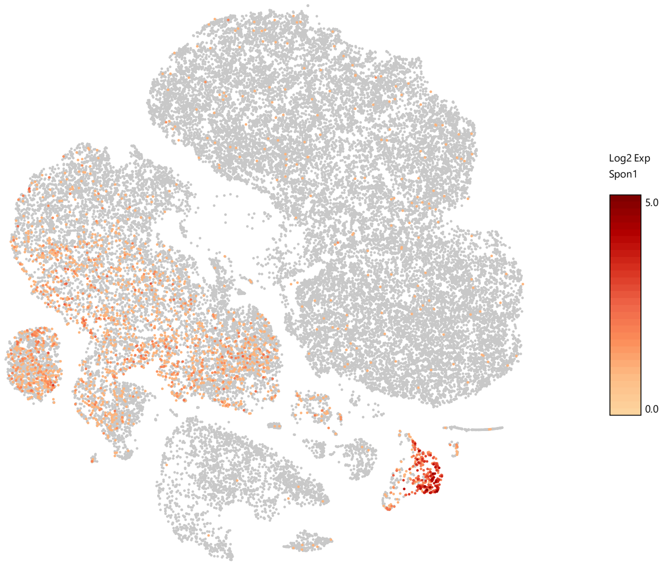

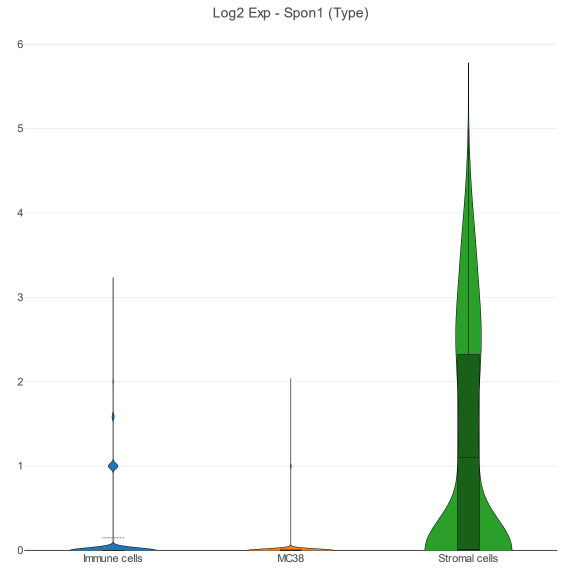

S1g

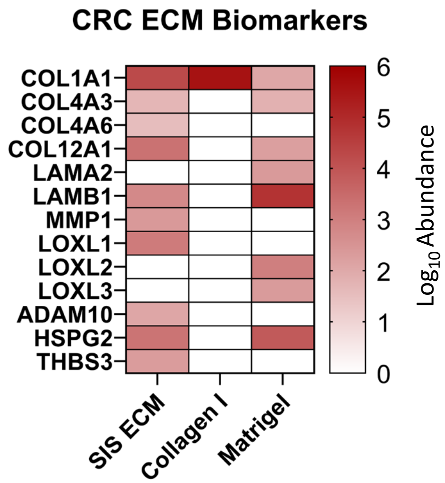
CRC ECM Biomarkers
COL1A1
COL4A3
COL4A6
COL12A1
LAMA2
LAMB1
MMP1
LOXL1
LOXL2
LOXL3
ADAM10
HSPG2
THBS3
SIS ECM
Collagen I
Matrigel
6
5
4
3
2
1
0
Log10 Abundance

Figure S1: a. Top 10 CRC core matrisome genes based upon bulk RNA Seq of cells alone spheroids at Day 7 of culture. b. Heatmap of core matrisome genes and their relative expression patterns. Expression data was normalized within each cell line column. c. Bar chart showing the overlap between core matrisome proteins identified within ECM biomaterials (LC-MS) vs. the number of CRC core matrisome genes identified from the cells alone spheroids. d. Percentage similarity between the ECM biomaterials and CRC spheroids. e. Core matrisome expression by cell type from in vivo MC38 tumors determined by scRNA Seq. f. tSNE and violin plots of relative expression of the top 5 matrisome genes predominantly expressed by non-cancer cells within in vivo MC38 tumors. g. Heatmap of CRC ECM biomarkers found within ECM biomaterials.

S2a

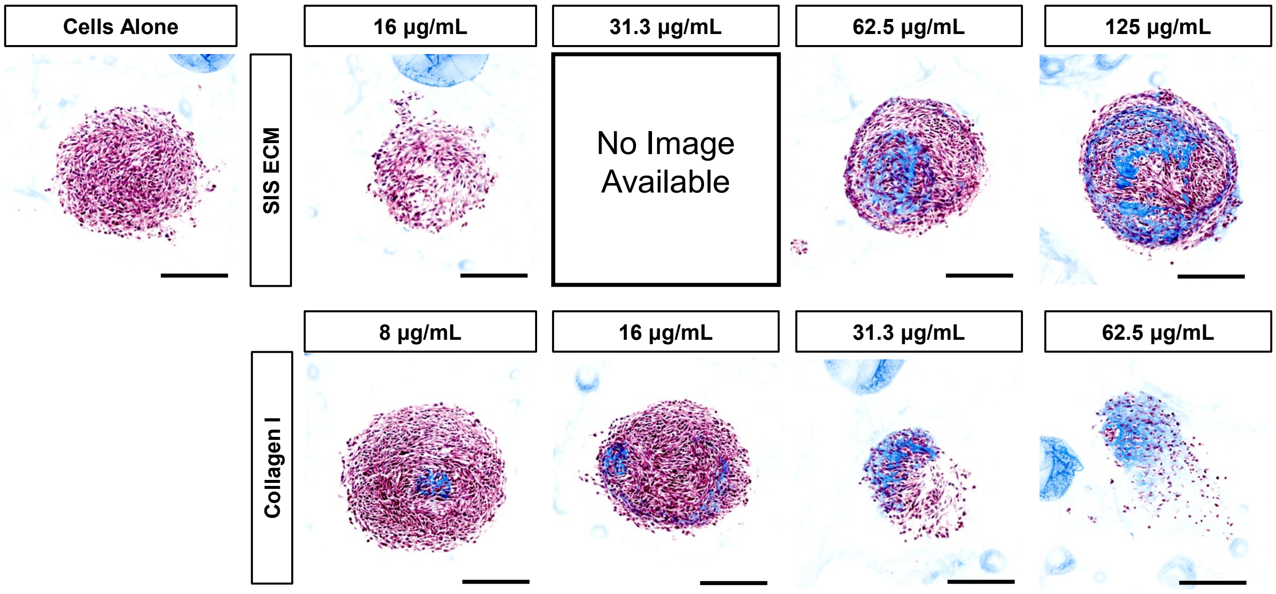
Cells Alone
16 µg/mL
31.3 µg/mL
62.5 µg/mL
125 µg/mL
SIS ECM
No Image Available
8 µg/mL
16 µg/mL
31.3 µg/mL
62.5 µg/mL
Collagen I

S2b

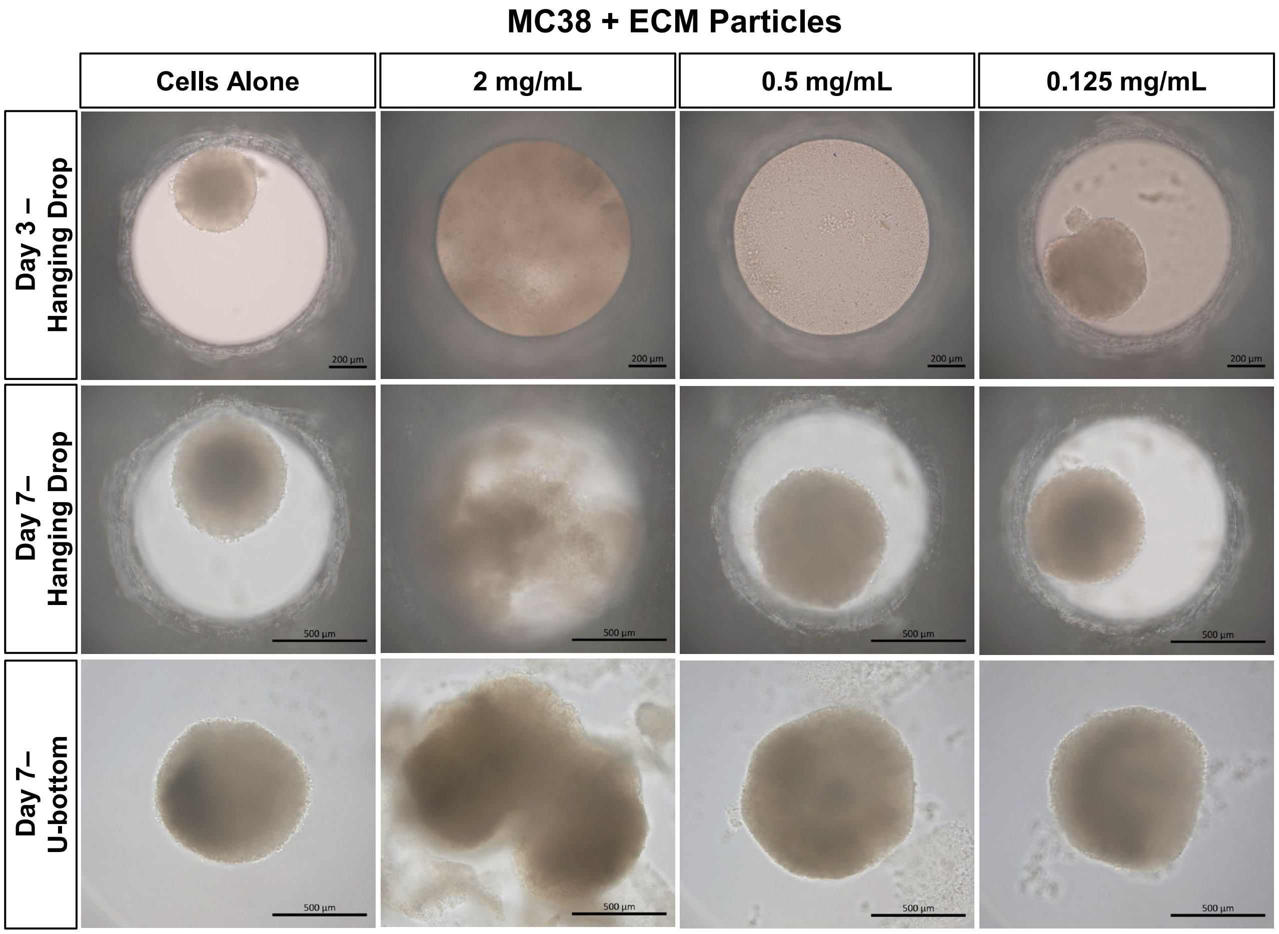
MC38 + ECM Particles
Cells Alone
2 mg/mL
0.5 mg/mL
0.125 mg/mL
Day 3 – Hanging Drop
Day 7– Hanging Drop
Day 7– U-bottom
200 µm
500 µm

S2c

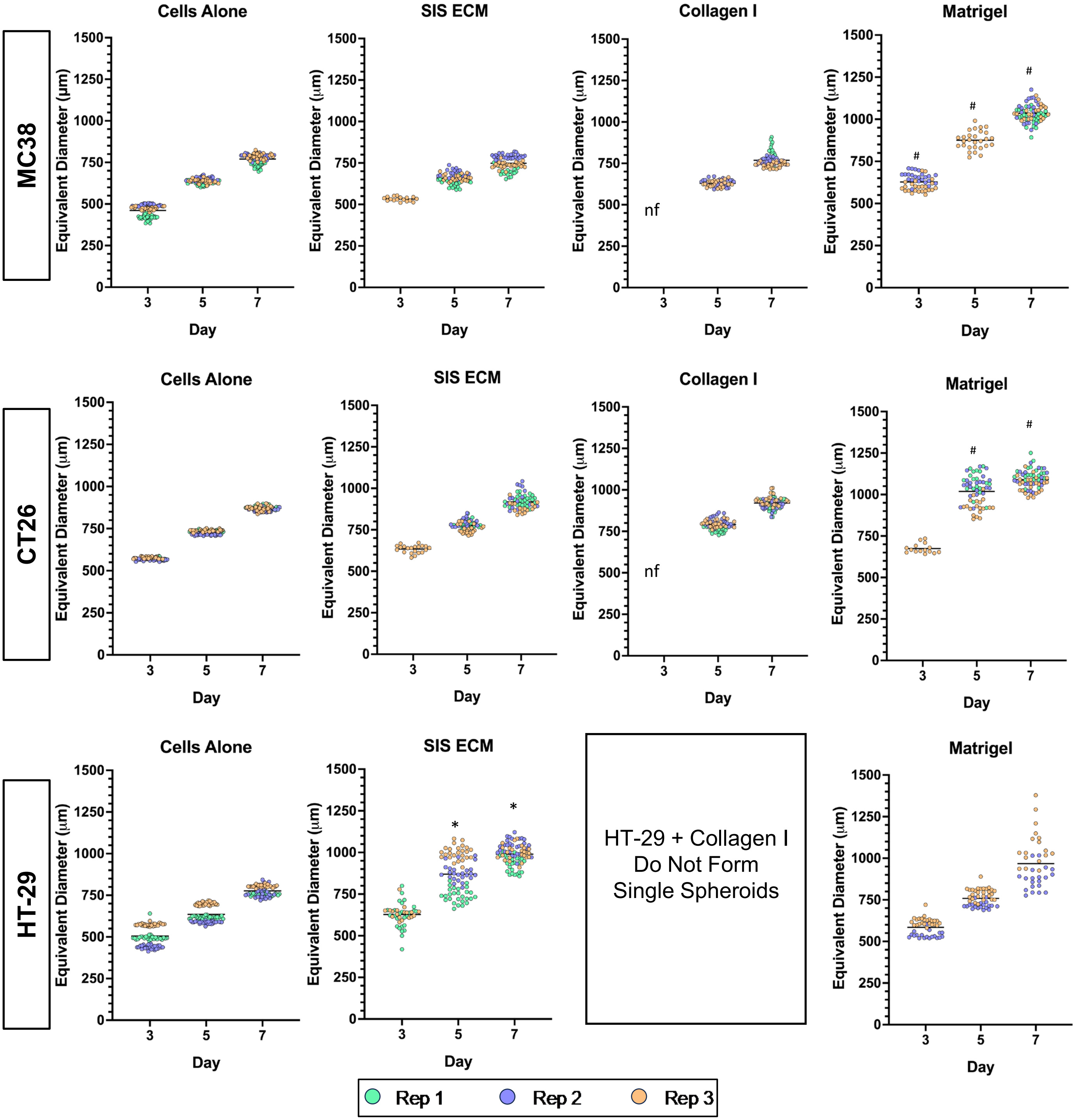

MC38
CT26
HT-29
Cells Alone
SIS ECM
Collagen I
Matrigel
Equivalent Diameter (µm)
Day
nf
HT-29 + Collagen I Do Not Form Single Spheroids
Rep 1
Rep 2
Rep 3

S2d

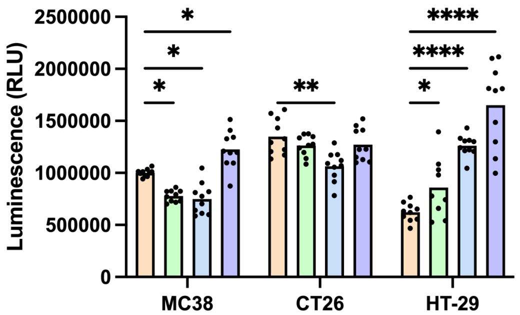

Luminescence (RLU)
2500000
2000000
1500000
1000000
500000
0
MC38
CT26
HT-29

S2e

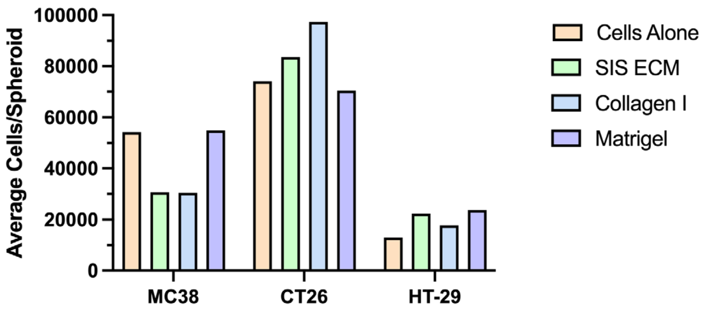

Average Cells/Spheroid
100000
80000
60000
40000
20000
0
MC38
CT26
HT-29
Cells Alone
SIS ECM
Collagen I
Matrigel

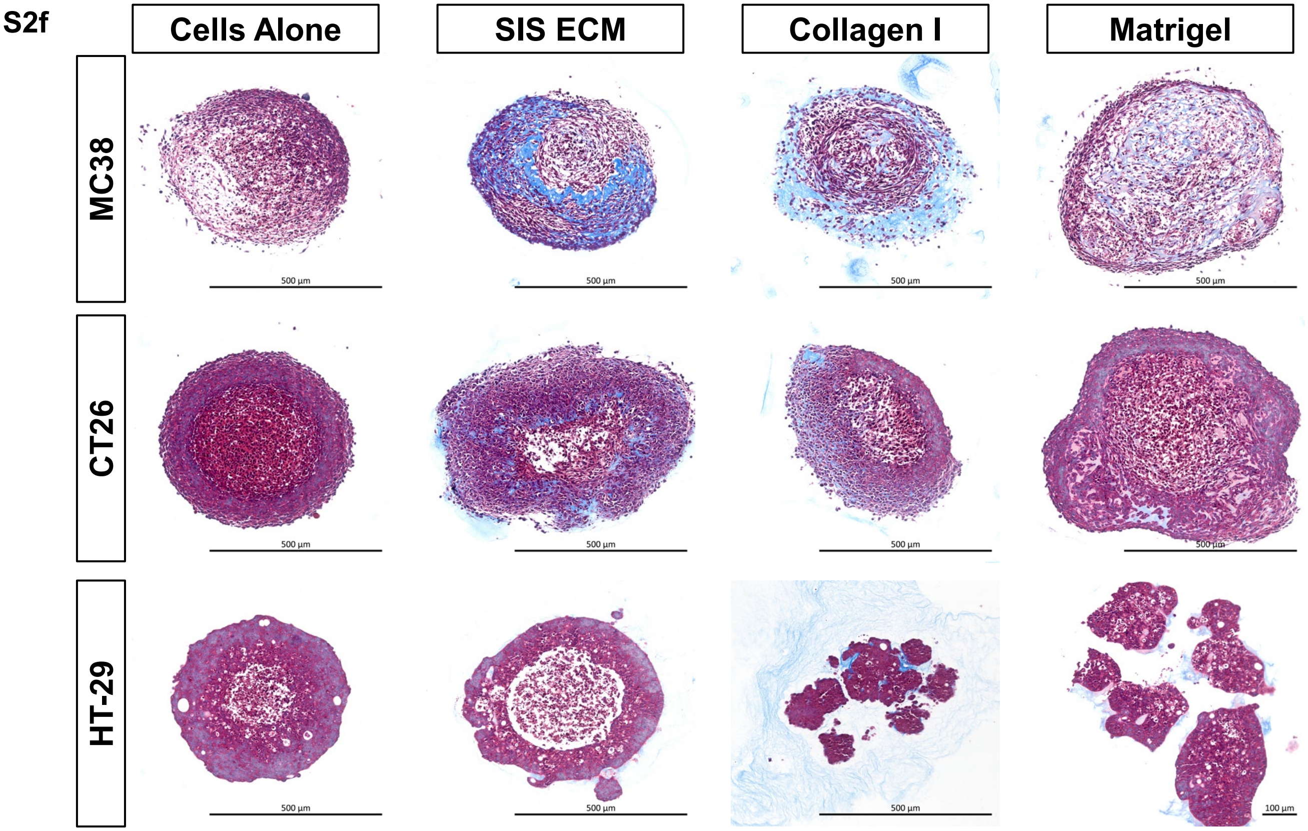
S2f
Cells Alone
SIS ECM
Collagen I
Matrigel
MC38
CT26
HT-29
500 µm
100 µm

Figure S2: a. Masson's Trichrome stained spheroid sections show varying concentrations of SIS ECM digest and Type I Collagen integrate within MC38 MatriSpheres. b. SIS ECM particles were combined with MC38 cells to form spheroids via hanging drop or ultra-low attachment round-bottom 96-well plates. c. Quantification of equivalent diameters at days 3, 5, and 7 of culture, n >= 37. Statistics are one-way ANOVA followed by Sídák's multiple comparisons test. # = Matrigel statistically different from cells alone on the same day, * = SIS ECM statistically different from cells alone on the same day. d. Quantification of metabolic activity via CellTiter-Glo 3D assay at day 7. Statistics were calculated by a two-way ANOVA followed by a Tukey's multiple comparisons test. e. Average number of cells per spheroid counted from manual dissociation of spheroids at day 7. f. Representative images of Masson's Trichrome stained spheroid sections at day 7. Scale bars are indicated on the images. Statistical significance where $p < 0.05$ is denoted with *, ≤ 0.01 with **, ≤ 0.001 with ***, and ≤ 0.0001 with ****.

S3a

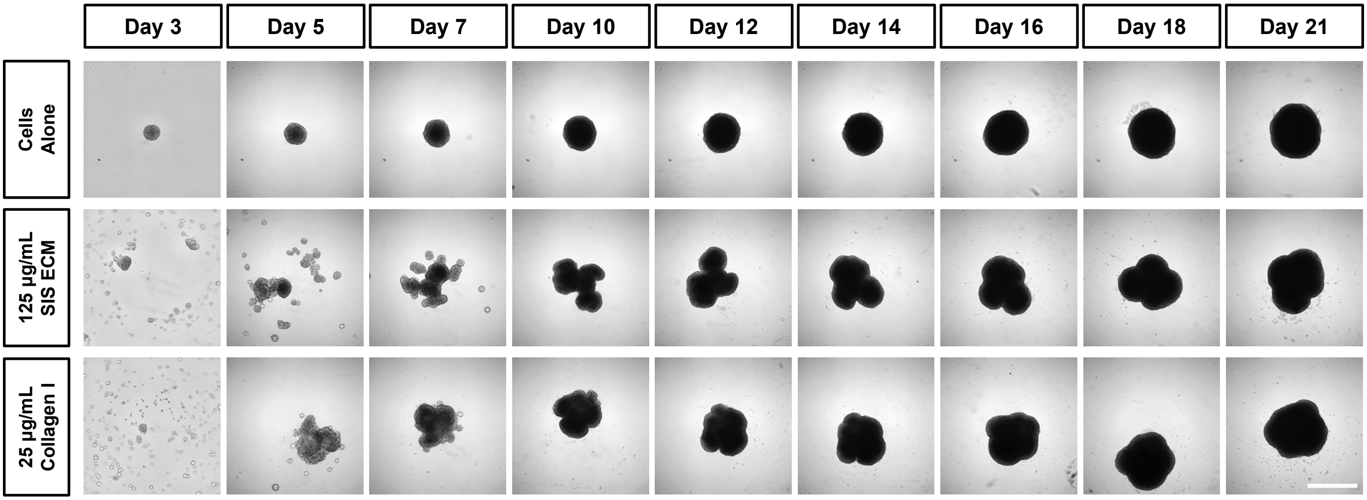

S3b

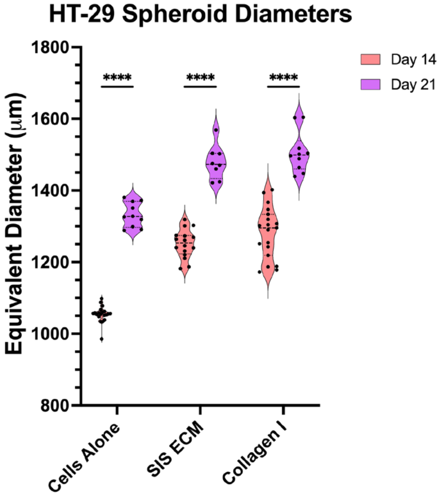

S3c

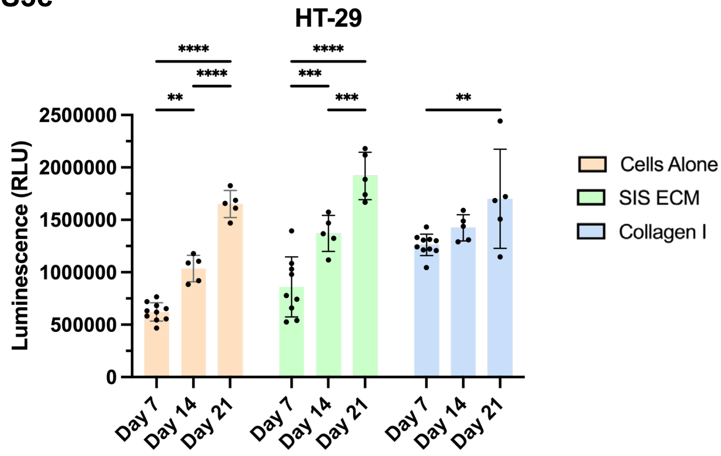

S3d

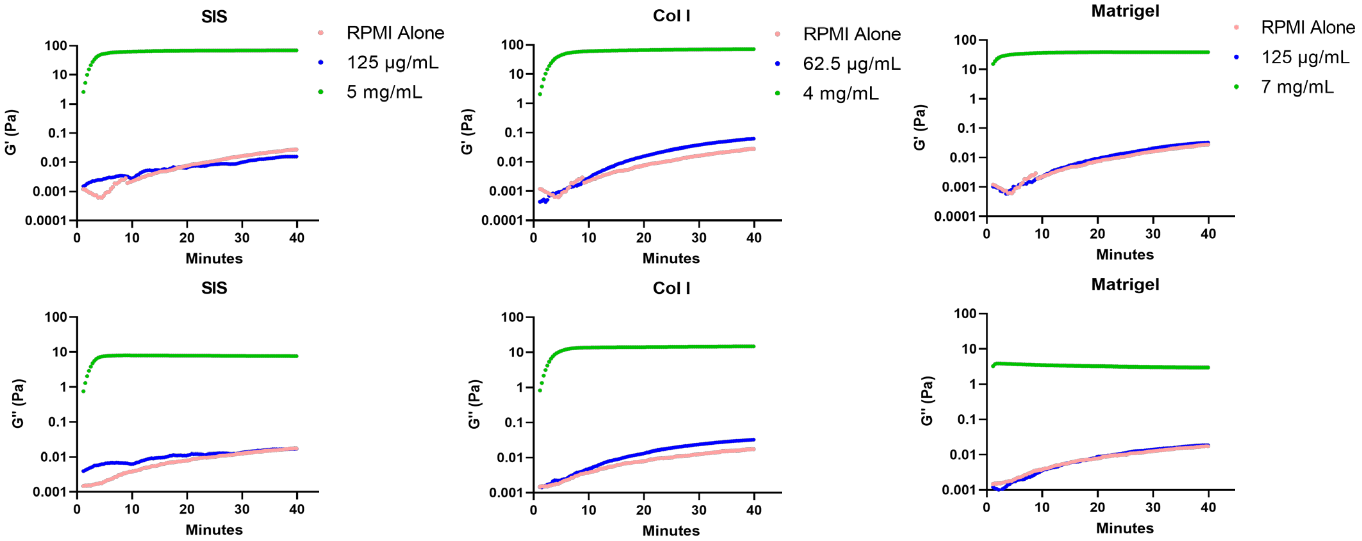

S3e

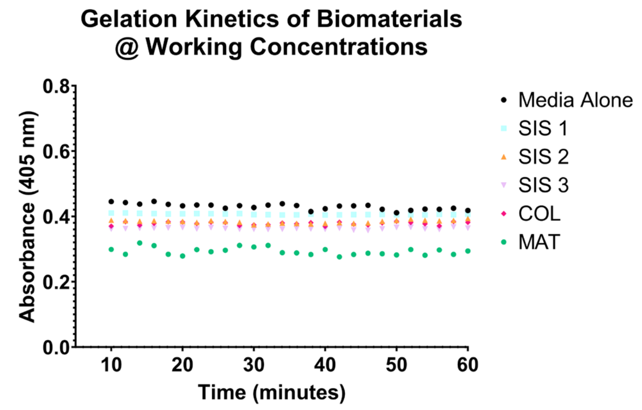
Gelation Kinetics of Biomaterials
@ Working Concentrations
Absorbance (405 nm)
0.0
0.2
0.4
0.6
0.8
10
20
30
40
50
60
Time (minutes)
Media Alone
SIS 1
SIS 2
SIS 3
COL
MAT

S3f

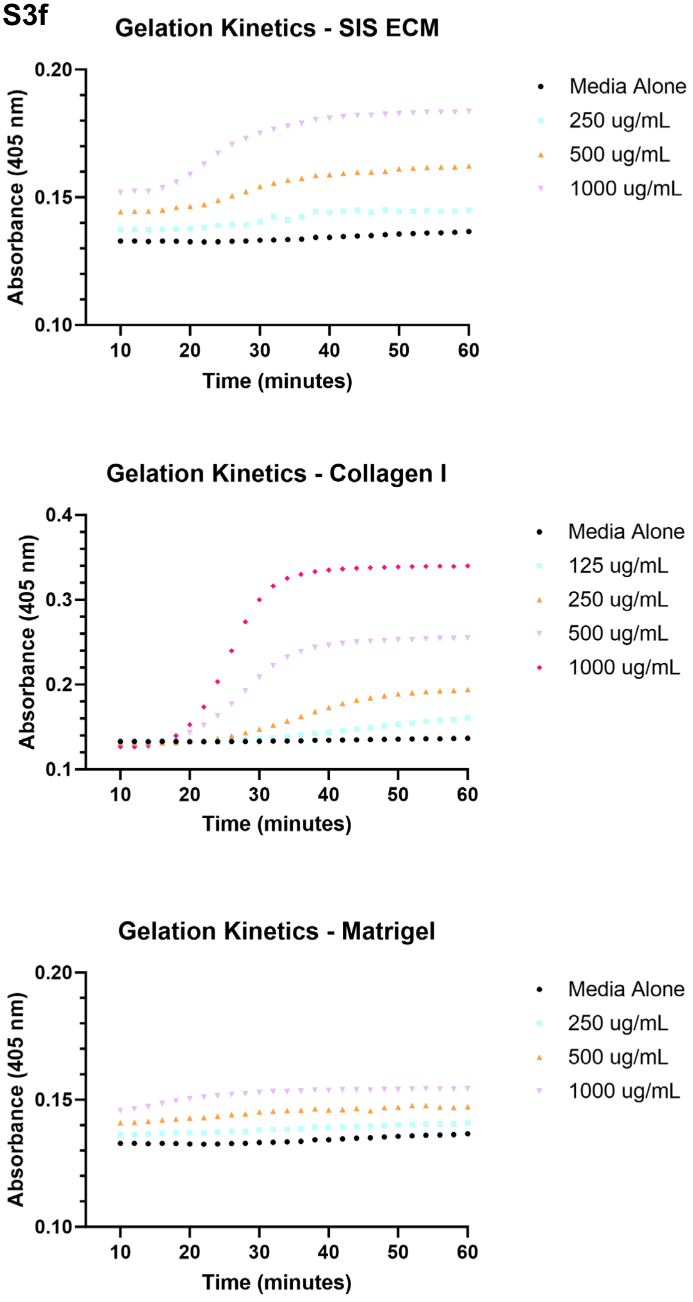
Gelation Kinetics - SIS ECM
Absorbance (405 nm)
0.10
0.15
0.20
10
20
30
40
50
60
Time (minutes)
Media Alone
250 ug/mL
500 ug/mL
1000 ug/mL
Gelation Kinetics - Collagen I
Absorbance (405 nm)
0.1
0.2
0.3
0.4
10
20
30
40
50
60
Time (minutes)
Media Alone
125 ug/mL
250 ug/mL
500 ug/mL
1000 ug/mL
Gelation Kinetics - Matrigel
Absorbance (405 nm)
0.10
0.15
0.20
10
20
30
40
50
60
Time (minutes)
Media Alone
250 ug/mL
500 ug/mL
1000 ug/mL

Figure S3: A. Representative images of HT-29 spheroids grown for 21 days. b. Quantification of HT-29 MatriSphere diameters at days 14 and 21. Statistics were calculated by a two-way ANOVA followed by a Sídák's multiple comparisons test. c. Quantification of metabolic activity of spheroids via CellTiter-Glo 3D. d. Rheological characterization of cell culture media and ECM biomaterials at experimental and traditional hydrogel formation concentrations. e. Gelation kinetics curves showing turbidity of ECM biomaterials at working ECM concentrations in comparison to control RPMI media. f. Gelation kinetics curves at varying ECM concentration by ECM biomaterial. Statistical significance where $p < 0.05$ is denoted with *, ≤ 0.01 with **, ≤ 0.001 with ***, and ≤ 0.0001 with ****.

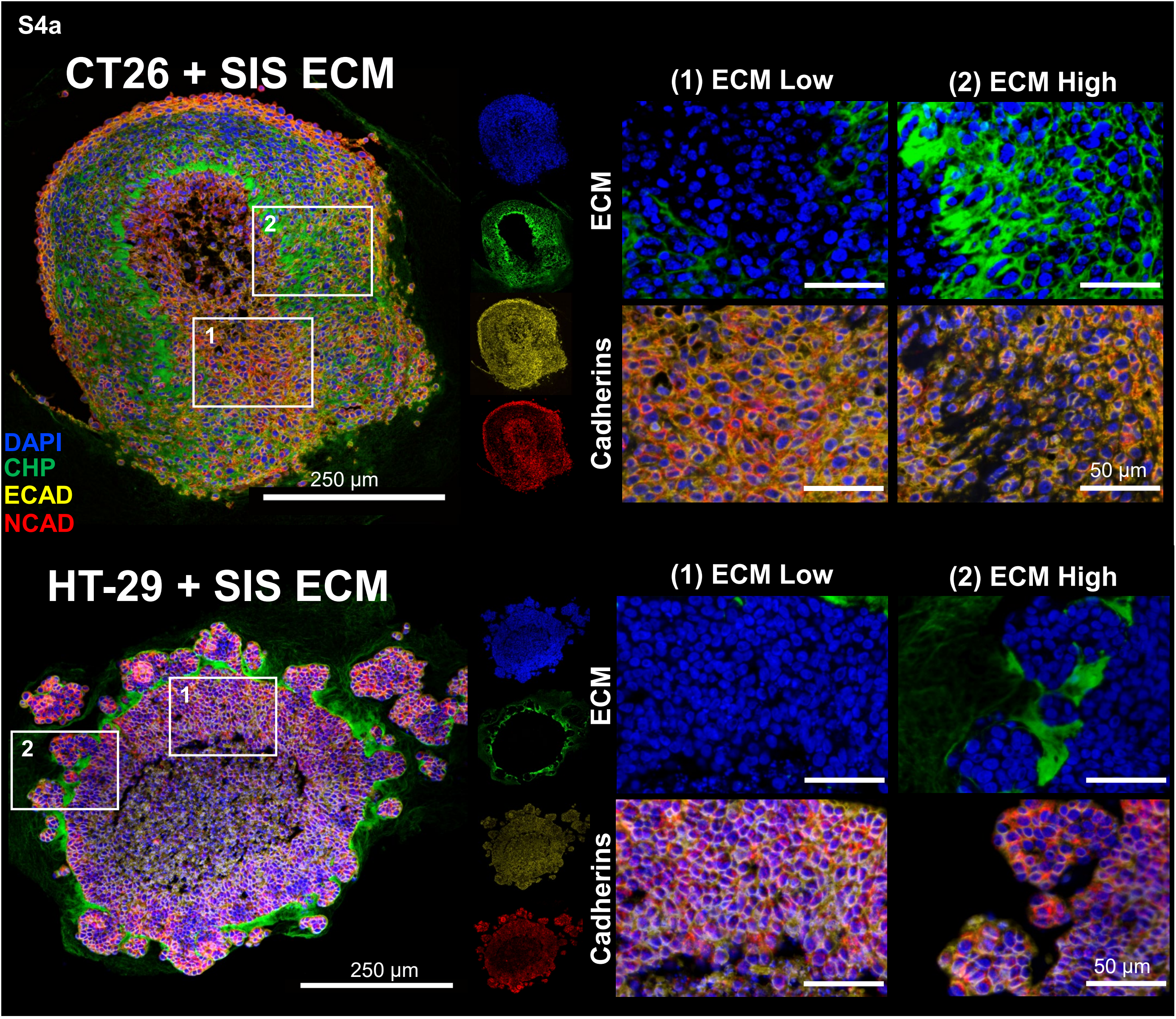
S4a
CT26 + SIS ECM
(1) ECM Low
(2) ECM High
ECM
Cadherins
DAPI
CHP
ECAD
NCAD
250 µm
50 µm
HT-29 + SIS ECM
(1) ECM Low
(2) ECM High
ECM
Cadherins
250 µm
50 µm

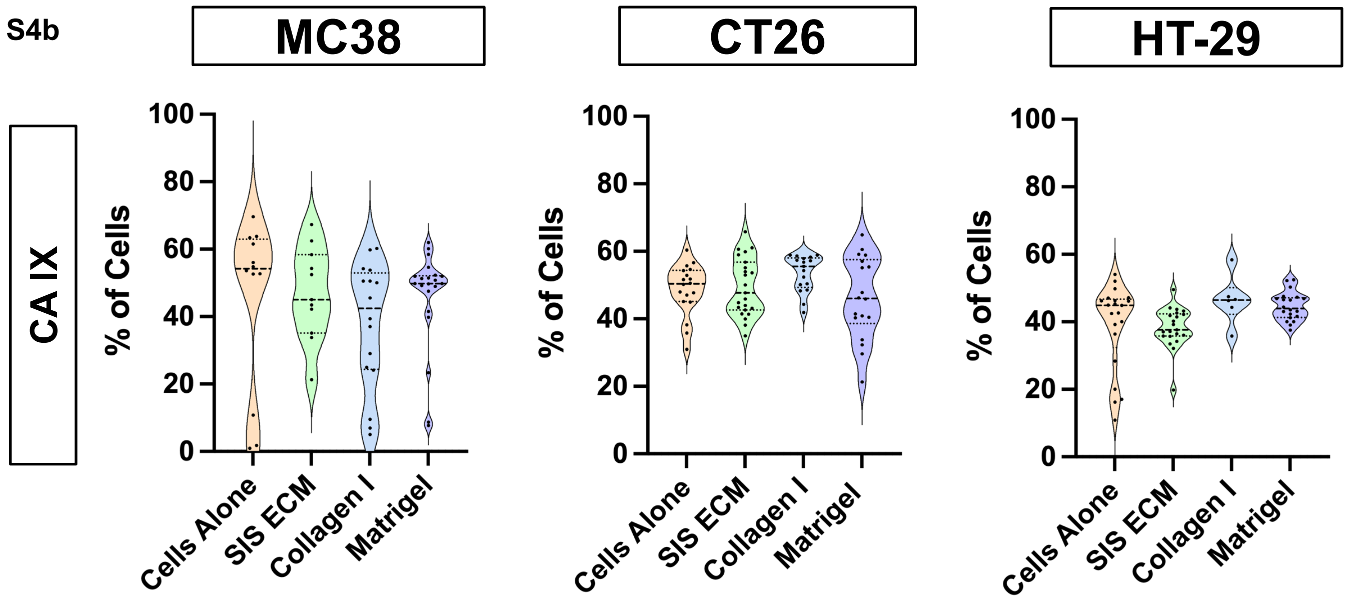
S4b
MC38
CT26
HT-29
CA IX
% of Cells
0
20
40
60
80
100
Cells Alone
SIS ECM
Collagen I
Matrigel

S4c

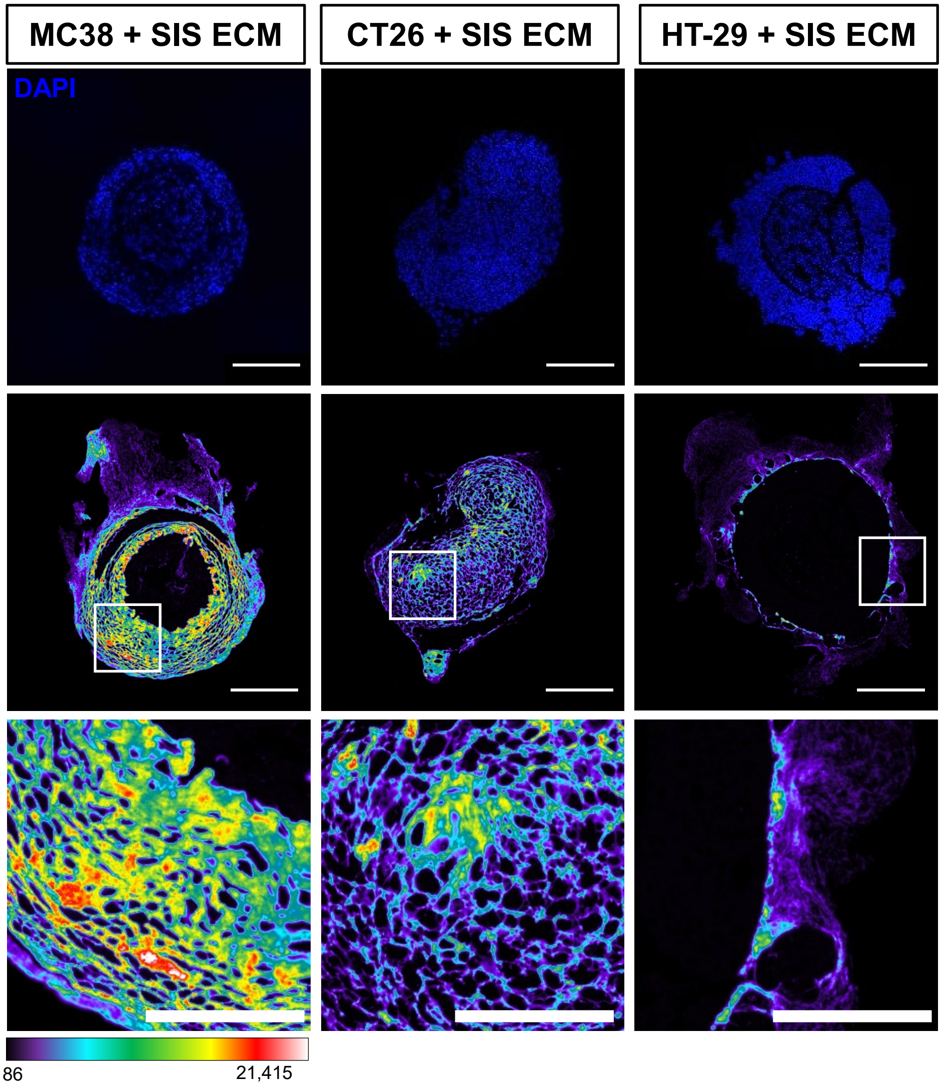

MC38

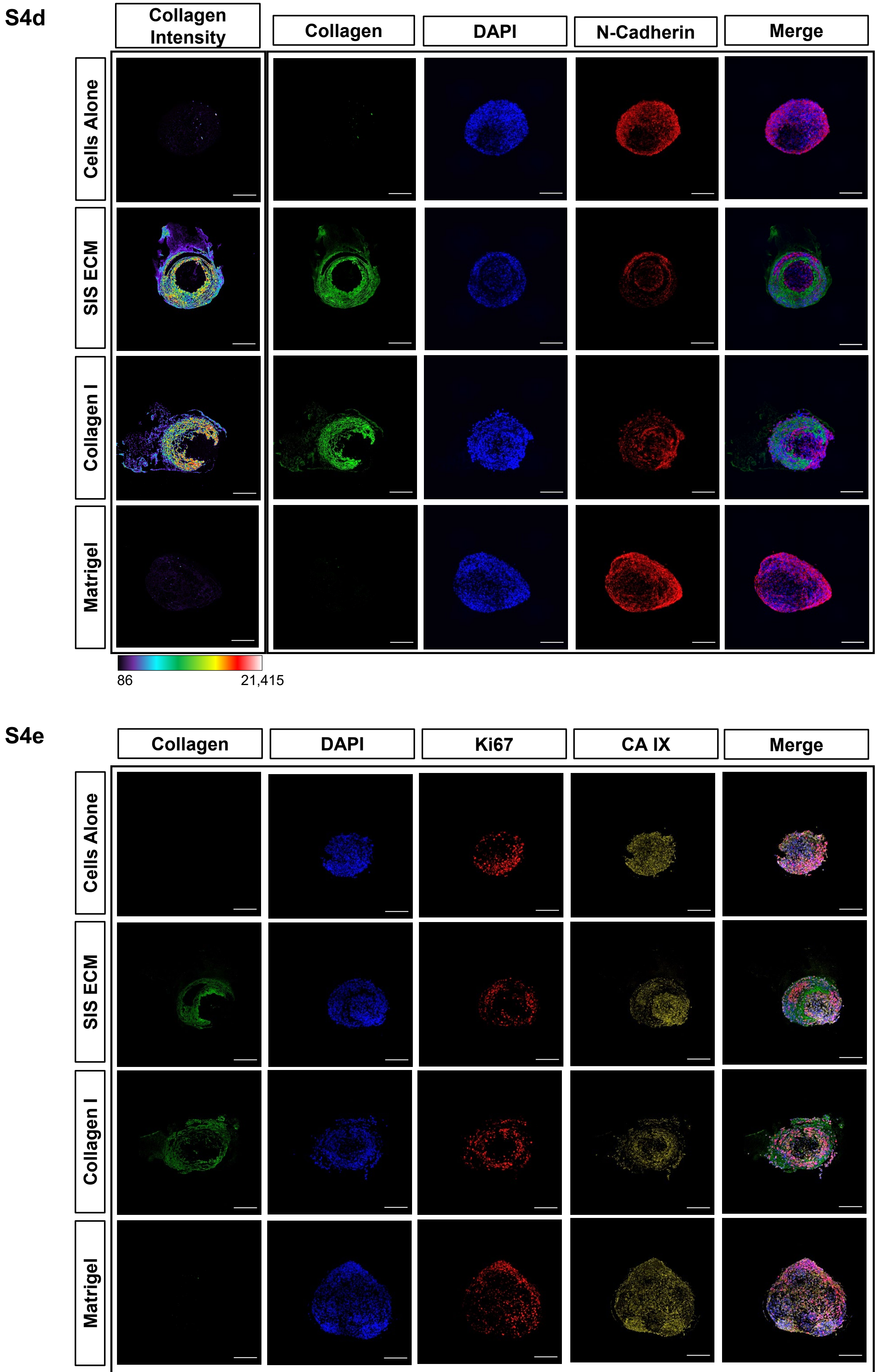
S4d
Collagen Intensity
Collagen
DAPI
N-Cadherin
Merge
Cells Alone
SIS ECM
Collagen I
Matrigel
86
21,415
S4e
Collagen
DAPI
Ki67
CA IX
Merge
Cells Alone
SIS ECM
Collagen I
Matrigel

CT26

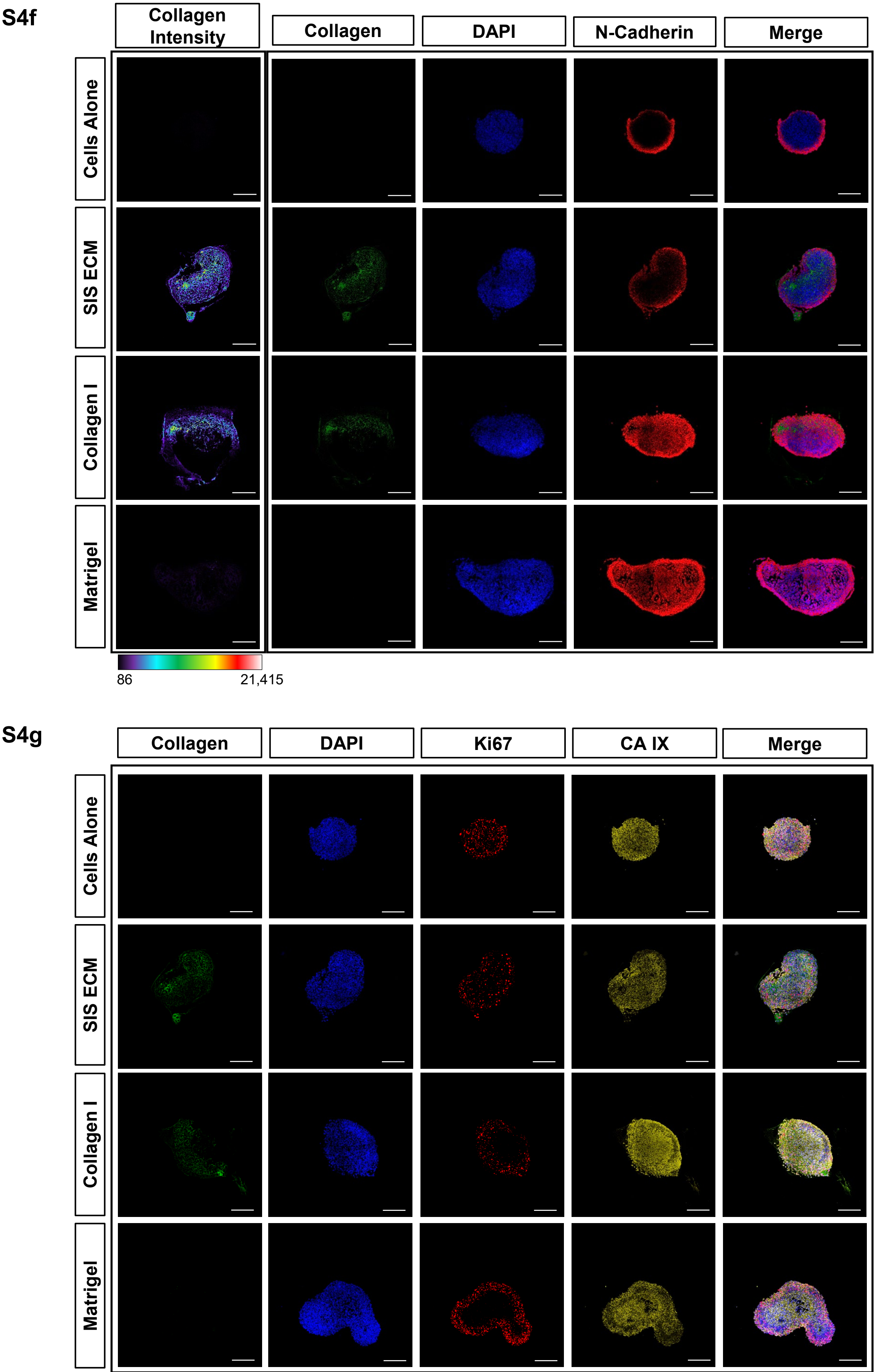
S4f
Collagen Intensity
Collagen
DAPI
N-Cadherin
Merge
Cells Alone
SIS ECM
Collagen I
Matrigel
86
21,415
S4g
Collagen
DAPI
Ki67
CA IX
Merge
Cells Alone
SIS ECM
Collagen I
Matrigel

HT-29

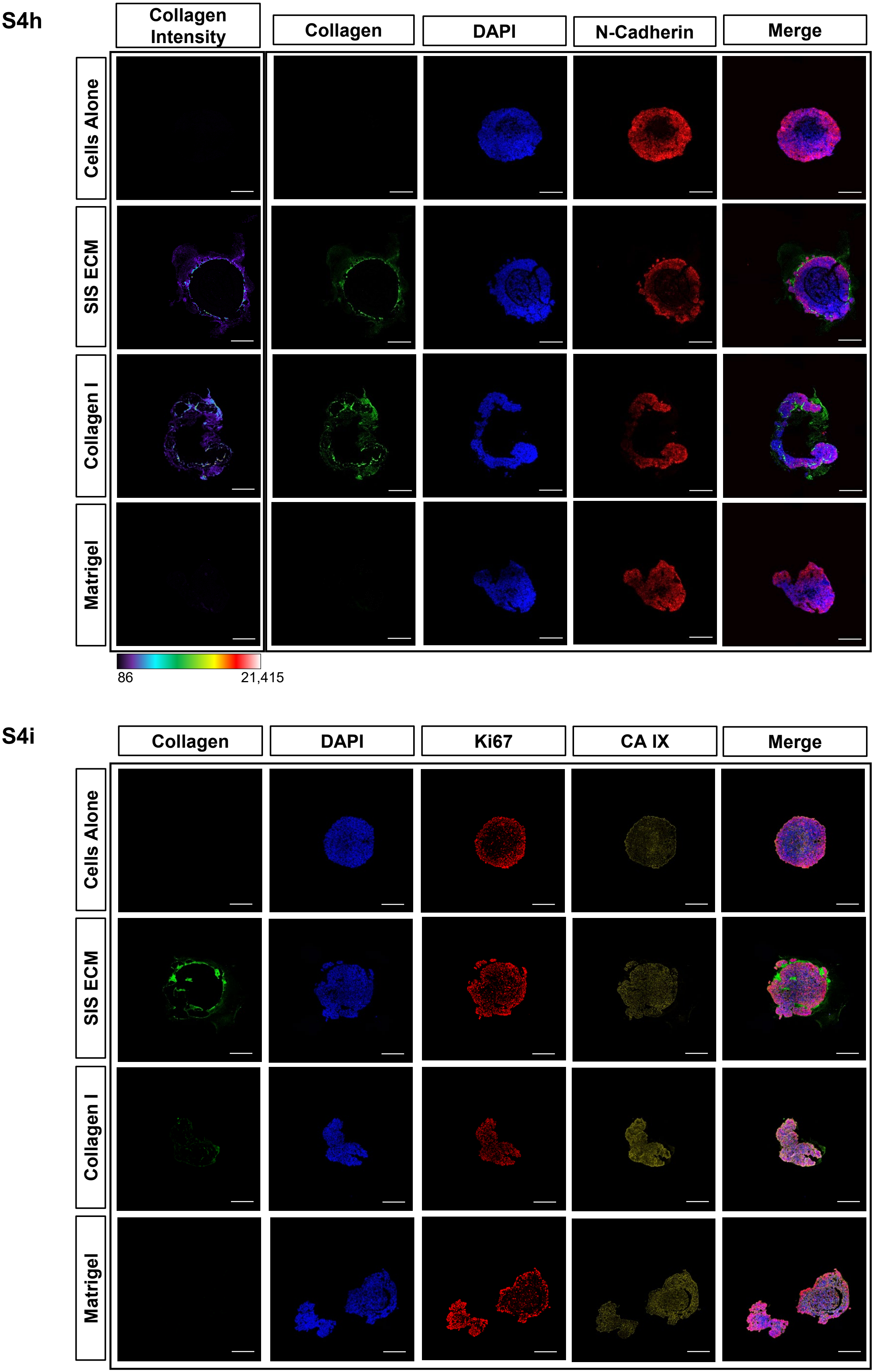
S4h
Collagen Intensity
Collagen
DAPI
N-Cadherin
Merge
Cells Alone
SIS ECM
Collagen I
Matrigel
86
21,415
S4i
Collagen
DAPI
Ki67
CA IX
Merge
Cells Alone
SIS ECM
Collagen I
Matrigel

Figure S4: a. Representative images of CT26 + SIS ECM & HT-29 + SIS ECM spheroid sections stained for E-CAD, N-CAD, CHP, and DAPI. b. Quantification of CA IX staining from representative spheroid sections. Statistics were calculated by a one-way ANOVA followed by a Tukey’s multiple comparisons test. c. Heatmaps of SIS ECM-incorporated spheroid sections stained with CHP to visualize denatured fibrillar collagen regions. d. Representative images of MC38 spheroid sections showing collagen intensity, CHP, DAPI, N-CAD, and the merged image. e. Representative images of MC38 spheroid sections showing, CHP, DAPI, Ki67, CA IX, and the merged image. f. Representative images of CT26 spheroid sections showing collagen intensity, CHP, DAPI, N-CAD, and the merged image. g. Representative images of CT26 spheroid sections showing, CHP, DAPI, Ki67, CA IX, and the merged image. h. Representative images of HT-29 spheroid sections showing collagen intensity, CHP, DAPI, N-CAD, and the merged image. i. Representative images of HT-29 spheroid sections showing, CHP, DAPI, Ki67, CA IX, and the merged image. Statistical significance where $p < 0.05$ is denoted with *, ≤ 0.01 with **, ≤ 0.001 with ***, and ≤ 0.0001 with ****.

S5a

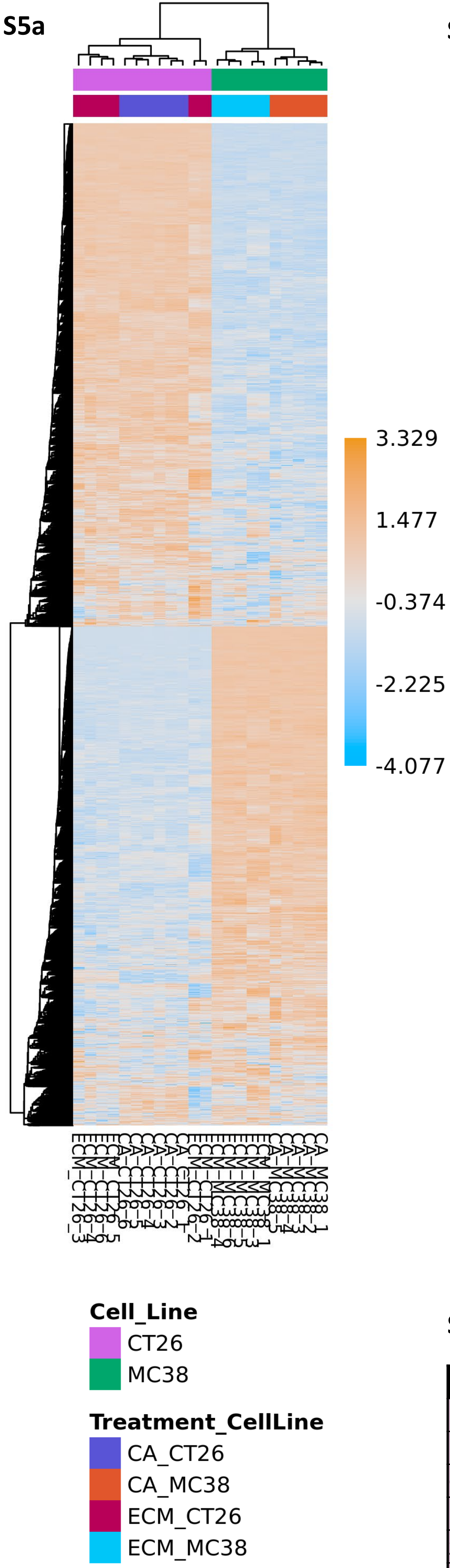

S5b

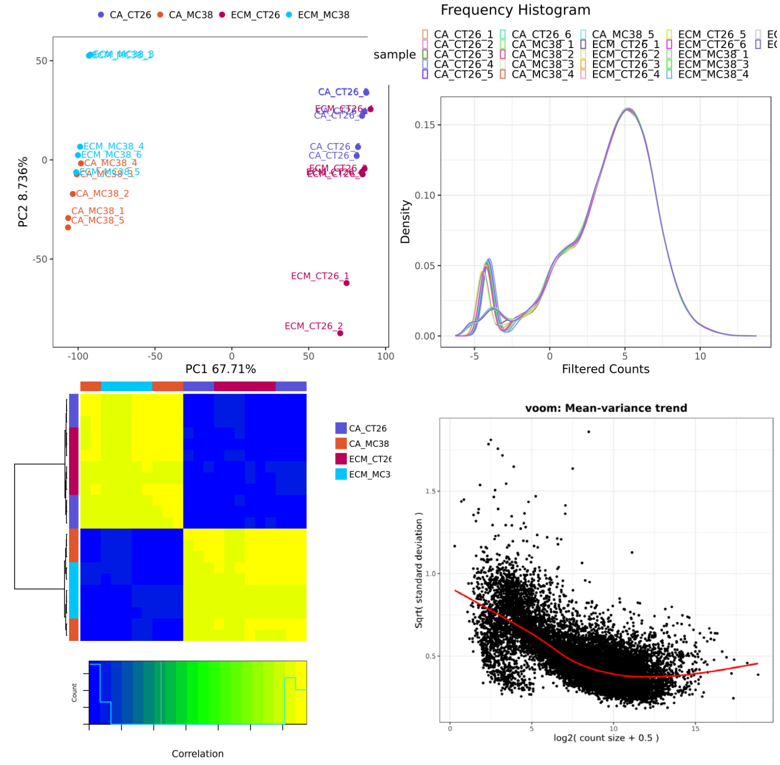

	ECM_MC38 -CA_MC38	ECM_CT26 -CA_CT26
upreg>1.2, pval<0.05	938	1066
downreg<-1.2, pval<0.05	1069	916
upreg>1.2, pval<0.01	580	764
downreg<-1.2, pval<0.01	667	576
upreg>1.2, adjpval<0.05	431	678
downreg<-1.2, adjpval<0.05	506	494
upreg>1.2, adjpval<0.01	178	418
downreg<-1.2, adjpval<0.01	216	262

Please note Sample size:
CA_CT26=6
CA_MC38=5
ECM_CT26=6
ECM_MC38=5

Contrasts:
ECM_MC38-CA_MC38 : c(ECM_MC38 = 5, CA_MC38 = 5)
ECM_CT26-CA_CT26 : c(ECM_CT26 = 6, CA_CT26 = 6)

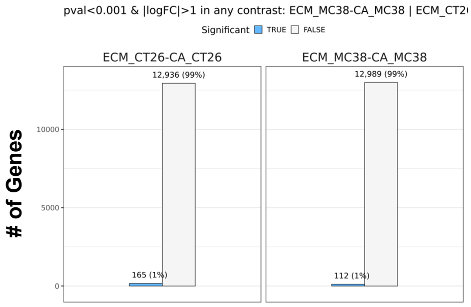

S5c

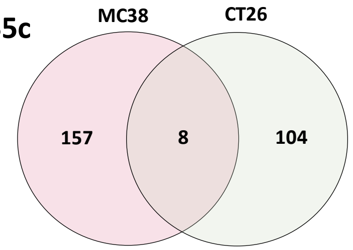

Genes Influenced by ECM	
Mmp13	St3gal4
Fam102a	Dlgap2
Gm8995	Dlx2
Ddx41	Aspn

S5d

	SIS ECM vs Cells Alone			
	MC38		CT26	
Genes	Fold Change	Adj p-value	Fold Change	Adj p-value
Mmp13	2.90	6.30E-03	14.22	2.42E-13
Fam102a	2.12	1.34E-08	2.61	8.98E-11
Gm8995	2.08	6.68E-03	2.72	4.71E-09
Ddx41	2.06	6.10E-08	2.00	1.91E-08
St3gal4	2.05	1.44E-08	2.09	9.51E-09
Dlgap2	2.03	2.54E-03	3.75	4.30E-03
Dlx2	-3.99	6.19E-04	-5.05	1.64E-04
Aspn	-6.13	5.29E-07	-2.01	7.96E-03

S5e

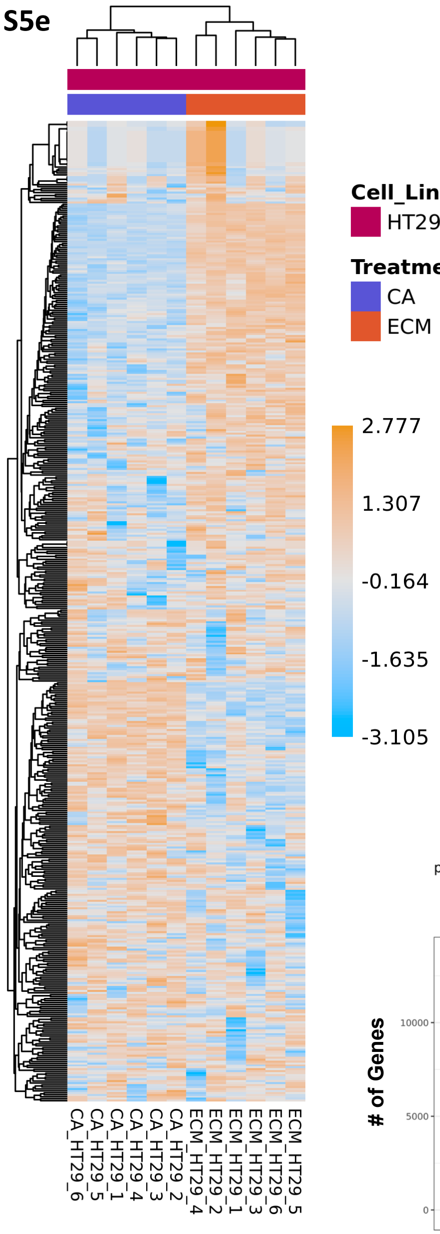

S5f

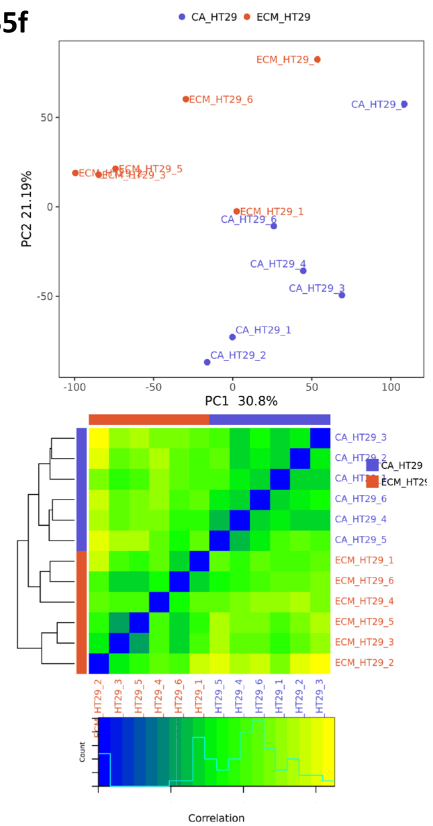

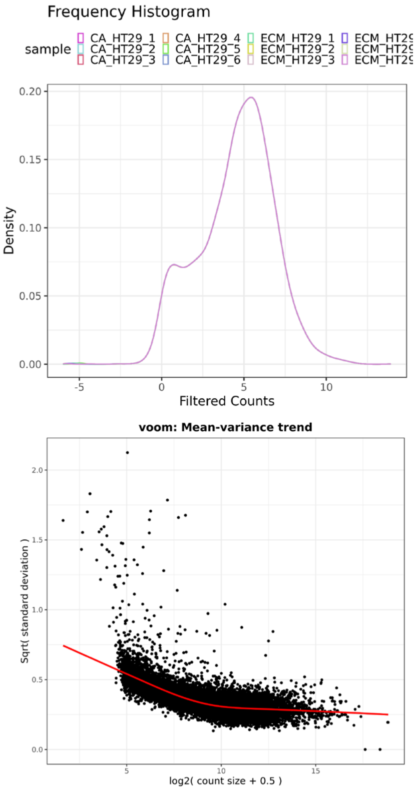

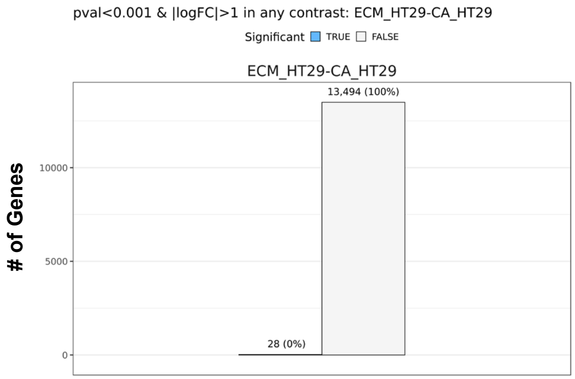

Please note Sample size:
CA_HT29=6
ECM_HT29=6

Contrasts:
ECM_HT29-CA_HT29 : c(ECM_HT29 = 6, CA_HT29 = 6)

	ECM_HT29 -CA_HT29
upreg>1.2, pval<0.05	753
downreg<-1.2, pval<0.05	544
upreg>1.2, pval<0.01	627
downreg<-1.2, pval<0.01	398
upreg>1.2, adjpval<0.05	613
downreg<-1.2, adjpval<0.05	387
upreg>1.2, adjpval<0.01	404
downreg<-1.2, adjpval<0.01	245

S5g

HALLMARK_HYPOXIA

H: hallmark gene sets, ECM_MC38−CA_MC38, ES=−0.6, NES=−2.68, pval=0.00041, padj=0.0011

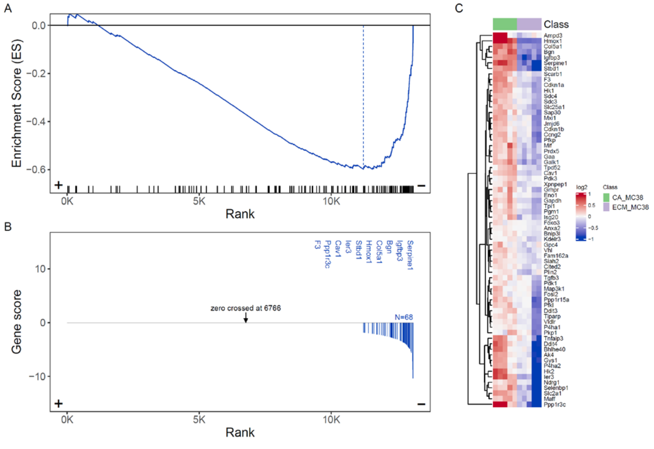

HALLMARK_INTERFERON_ALPHA_RESPONSE

H: hallmark gene sets, ECM_CT26−CA_CT26, ES=0.76, NES=3.03, pval=0.00038, padj=0.0024

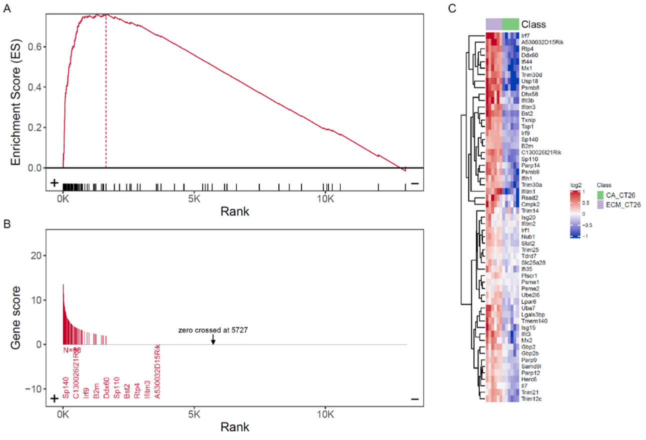

HALLMARK_INTERFERON_ALPHA_RESPONSE

H: hallmark gene sets, ECM_HT29−CA_HT29, ES=0.8, NES=3.21, pval=0.00036, padj=0.0014

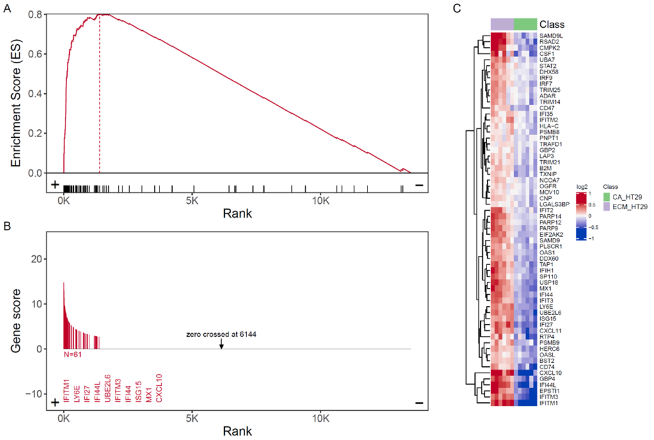

Figure S5: a. Heatmap showing normalized gene expression and hierarchical clustering of mouse CRC lines (MC38 and CT26) by sample. b. PCA plot, frequency histogram and number of mouse genes across different significant thresholds. c. Venn diagram of significantly overlapping genes between MC38 and CT26 d. table of corresponding fold change and adj. p-value of each gene. e. Heatmap showing normalized gene expression and hierarchical clustering of human CRC line HT-29 by sample. f. PCA plot, frequency histogram and number of human genes across different significant thresholds g. GSEA plots showing the top gene set within each cell line based upon enrichment score (ES). ES and gene score plots showing a heatmap of the leading-edge genes between treatments (SIS ECM vs. cells alone spheroids).

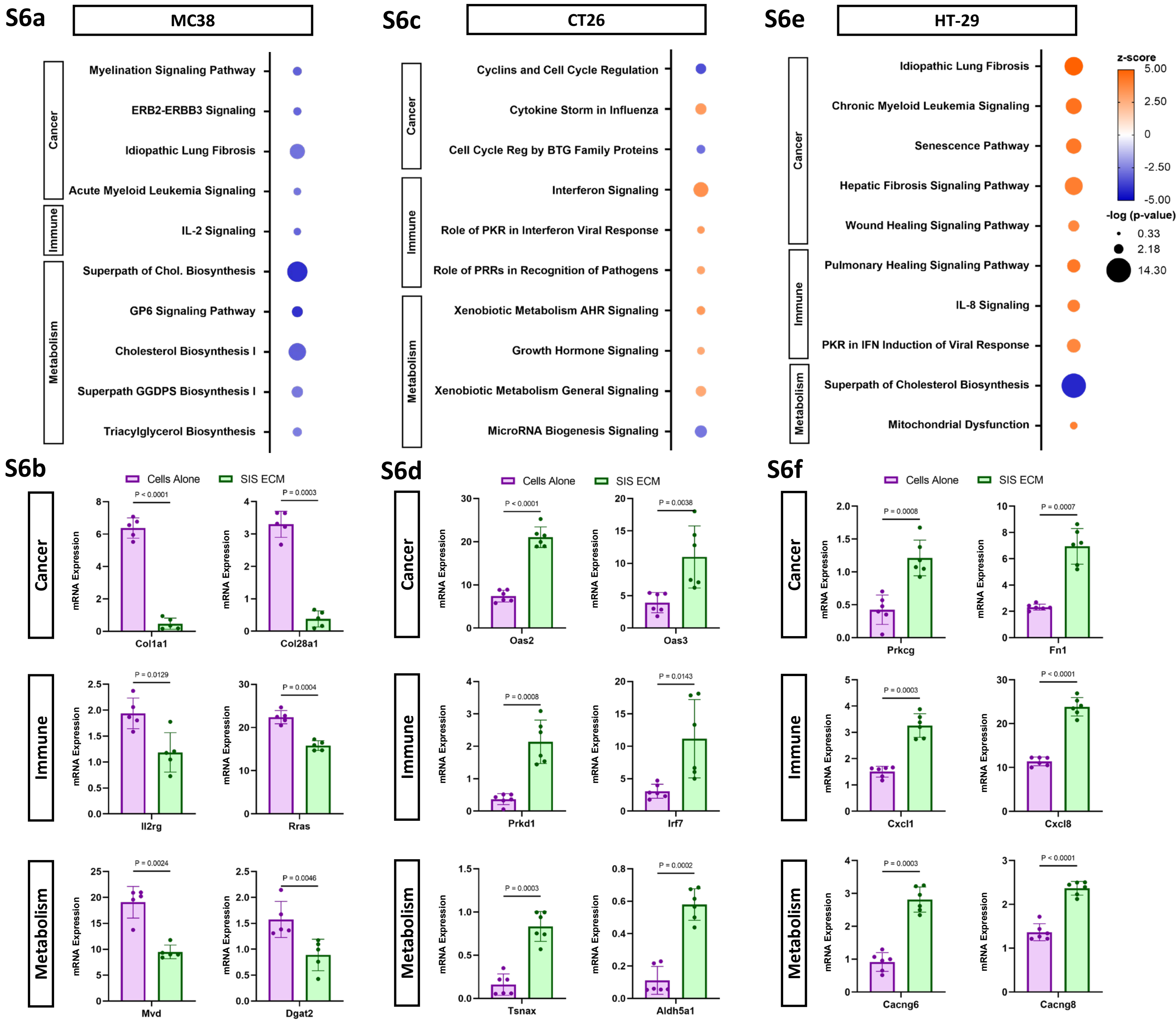
S6a
MC38
Cancer
Immune
Metabolism
Myelination Signaling Pathway
ERB2-ERBB3 Signaling
Idiopathic Lung Fibrosis
Acute Myeloid Leukemia Signaling
IL-2 Signaling
Superpath of Chol. Biosynthesis
GP6 Signaling Pathway
Cholesterol Biosynthesis I
Superpath GGDPS Biosynthesis I
Triacylglycerol Biosynthesis
S6c
CT26
Cyclins and Cell Cycle Regulation
Cytokine Storm in Influenza
Cell Cycle Reg by BTG Family Proteins
Interferon Signaling
Role of PKR in Interferon Viral Response
Role of PRRs in Recognition of Pathogens
Xenobiotic Metabolism AHR Signaling
Growth Hormone Signaling
Xenobiotic Metabolism General Signaling
MicroRNA Biogenesis Signaling
S6e
HT-29
Idiopathic Lung Fibrosis
Chronic Myeloid Leukemia Signaling
Senescence Pathway
Hepatic Fibrosis Signaling Pathway
Wound Healing Signaling Pathway
Pulmonary Healing Signaling Pathway
IL-8 Signaling
PKR in IFN Induction of Viral Response
Superpath of Cholesterol Biosynthesis
Mitochondrial Dysfunction
z-score
5.00
2.50
0
-2.50
-5.00
-log (p-value)
0.33
2.18
14.30
S6b
Cells Alone
SIS ECM
mRNA Expression
P < 0.0001
Col1a1
P = 0.0003
Col28a1
P = 0.0129
Il2rg
P = 0.0004
Rras
P = 0.0024
Mvd
P = 0.0046
Dgat2
S6d
P < 0.0001
Oas2
P = 0.0038
Oas3
P = 0.0008
Prkd1
P = 0.0143
Irf7
P = 0.0003
Tsnax
P = 0.0002
Aldh5a1
S6f
P = 0.0008
Prkcg
P = 0.0007
Fn1
P = 0.0003
Cxcl1
P < 0.0001
Cxcl8
P = 0.0003
Cacng6
P < 0.0001
Cacng8

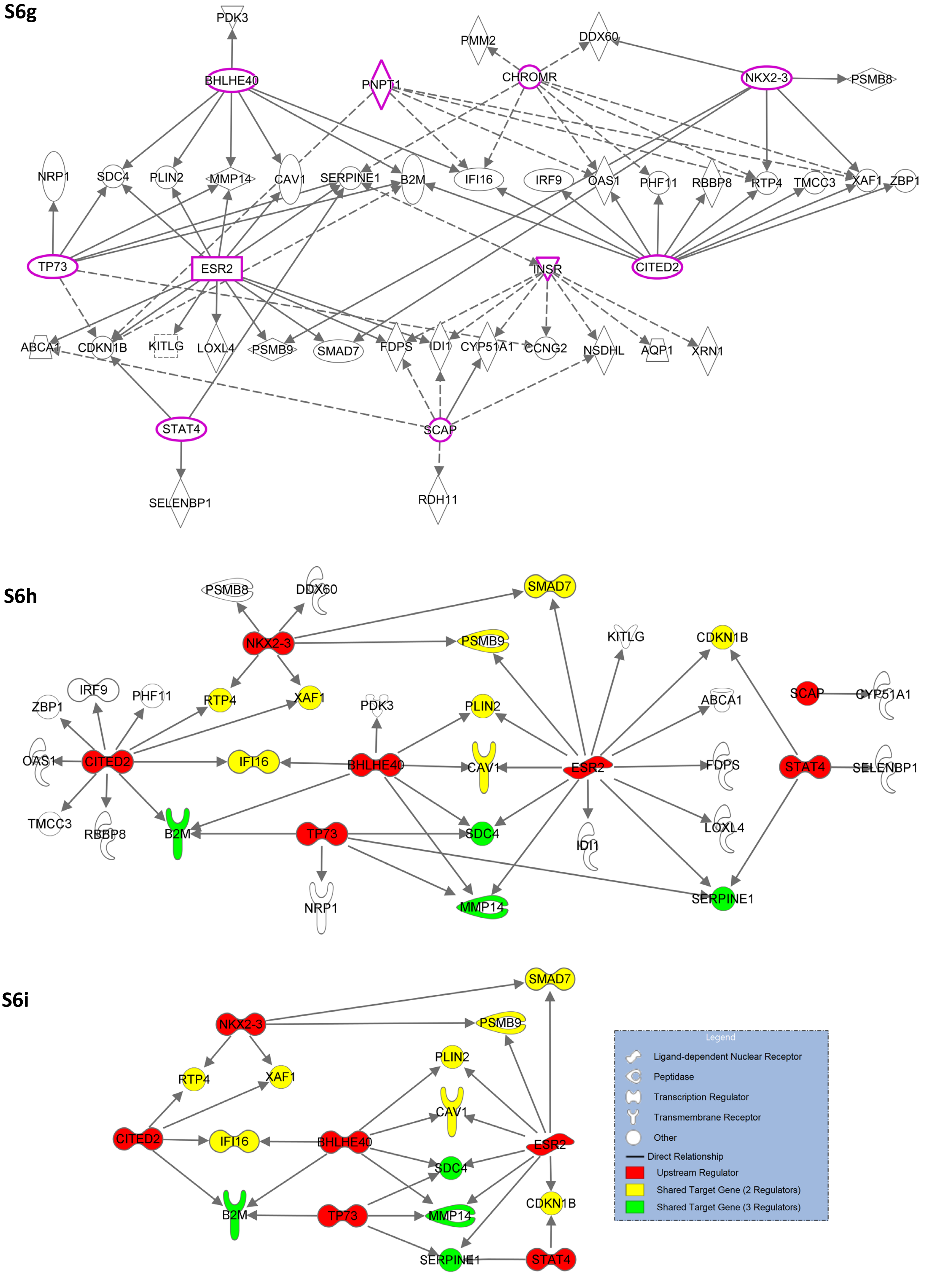
S6g
PDK3
PMM2
DDX60
BHLHE40
PNPT1
CHROMR
NKX2-3
PSMB8
NRP1
SDC4
PLIN2
MMP14
CAV1
SERPINE1
B2M
IFI16
IRF9
OAS1
PHF11
RBBP8
RTP4
TMCC3
XAF1
ZBP1
TP73
ESR2
INSR
CITED2
ABCA1
CDKN1B
KITLG
LOXL4
PSMB9
SMAD7
FDPS
IDI1
CYP51A1
CCNG2
NSDHL
AQP1
XRN1
STAT4
SCAP
SELENBP1
RDH11
S6h
PSMB8
DDX60
SMAD7
NKX2-3
PSMB9
KITLG
CDKN1B
IRF9
ZBP1
PHF11
RTP4
XAF1
PDK3
PLIN2
ABCA1
SCAP
CYP51A1
OAS1
CITED2
IFI16
BHLHE40
CAV1
ESR2
FDPS
STAT4
SELENBP1
TMCC3
RBBP8
B2M
TP73
SDC4
IDI1
LOXL4
NRP1
MMP14
SERPINE1
S6i
SMAD7
NKX2-3
PSMB9
PLIN2
RTP4
XAF1
CAV1
CITED2
IFI16
BHLHE40
ESR2
SDC4
B2M
TP73
MMP14
CDKN1B
SERPINE1
STAT4
Legend
Ligand-dependent Nuclear Receptor
Peptidase
Transcription Regulator
Transmembrane Receptor
Other
Direct Relationship
Upstream Regulator
Shared Target Gene (2 Regulators)
Shared Target Gene (3 Regulators)

S6j

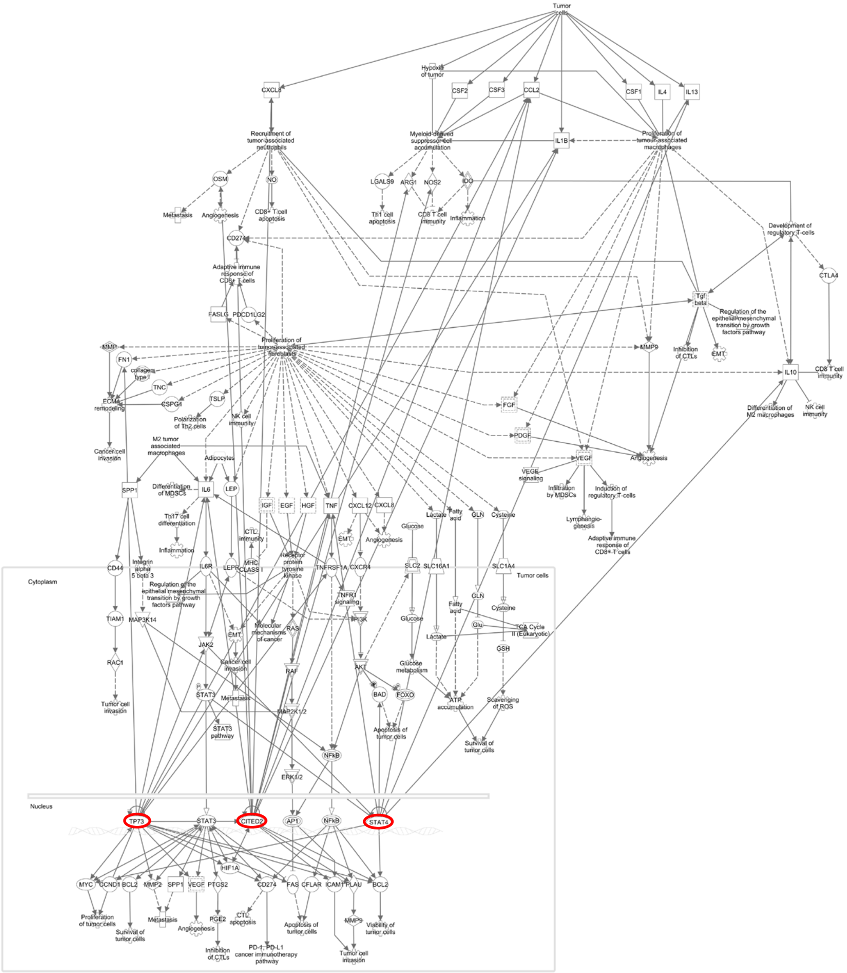

S6k

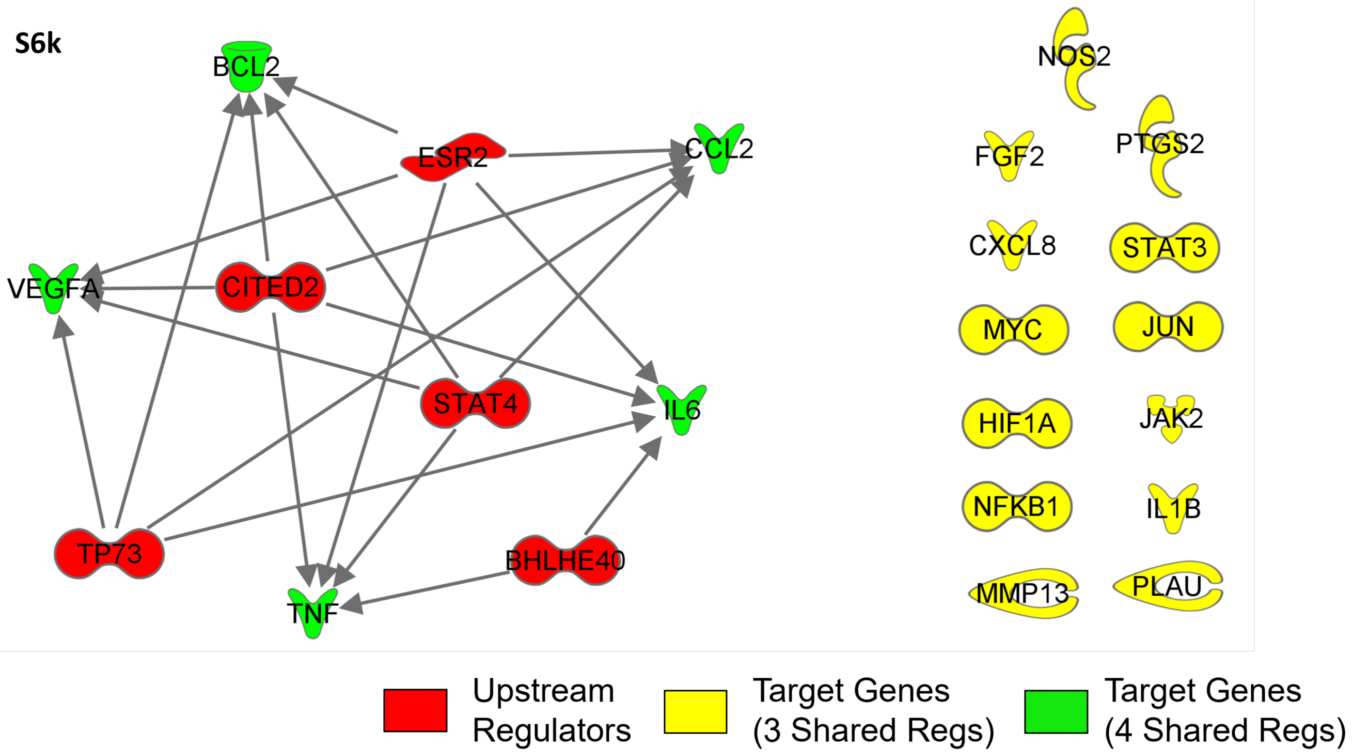
BCL2
ESR2
CCL2
VEGFA
CITED2
STAT4
IL6
TP73
BHLHE40
TNF
NOS2
FGF2
PTGS2
CXCL8
STAT3
MYC
JUN
HIF1A
JAK2
NFKB1
IL1B
MMP13
PLAU
Upstream Regulators
Target Genes (3 Shared Regs)
Target Genes (4 Shared Regs)

Figure S6: Bubble plots of the top 10 IPA predicted activation and top 2 genes identified within the assigned categories by cell line a-b. MC38 c-d. CT26 e-f. HT-29 g. All conserved upstream regulators and their downstream targets across CRC cell lines predicted by IPA. h. Only direct interactions between upstream regulators and target genes i. Direct interactions between gene targets with multiple shared regulators j. IPA tumor microenvironment pathway showing an example of interaction between target genes with 3 identified upstream regulators k. Interaction network between the identified upstream regulators and their most common targets within the TME pathways.

S7

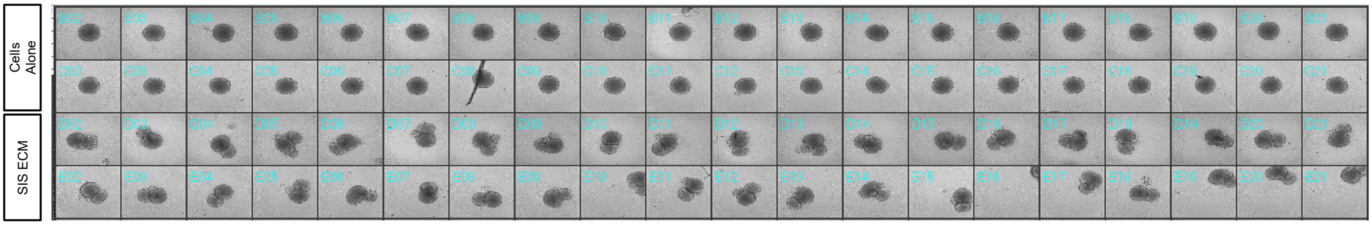

Figure S7: Representative images of HT-29 cells alone spheroids and MatriSpheres cultured in 384-well plates for 7 days.

S8

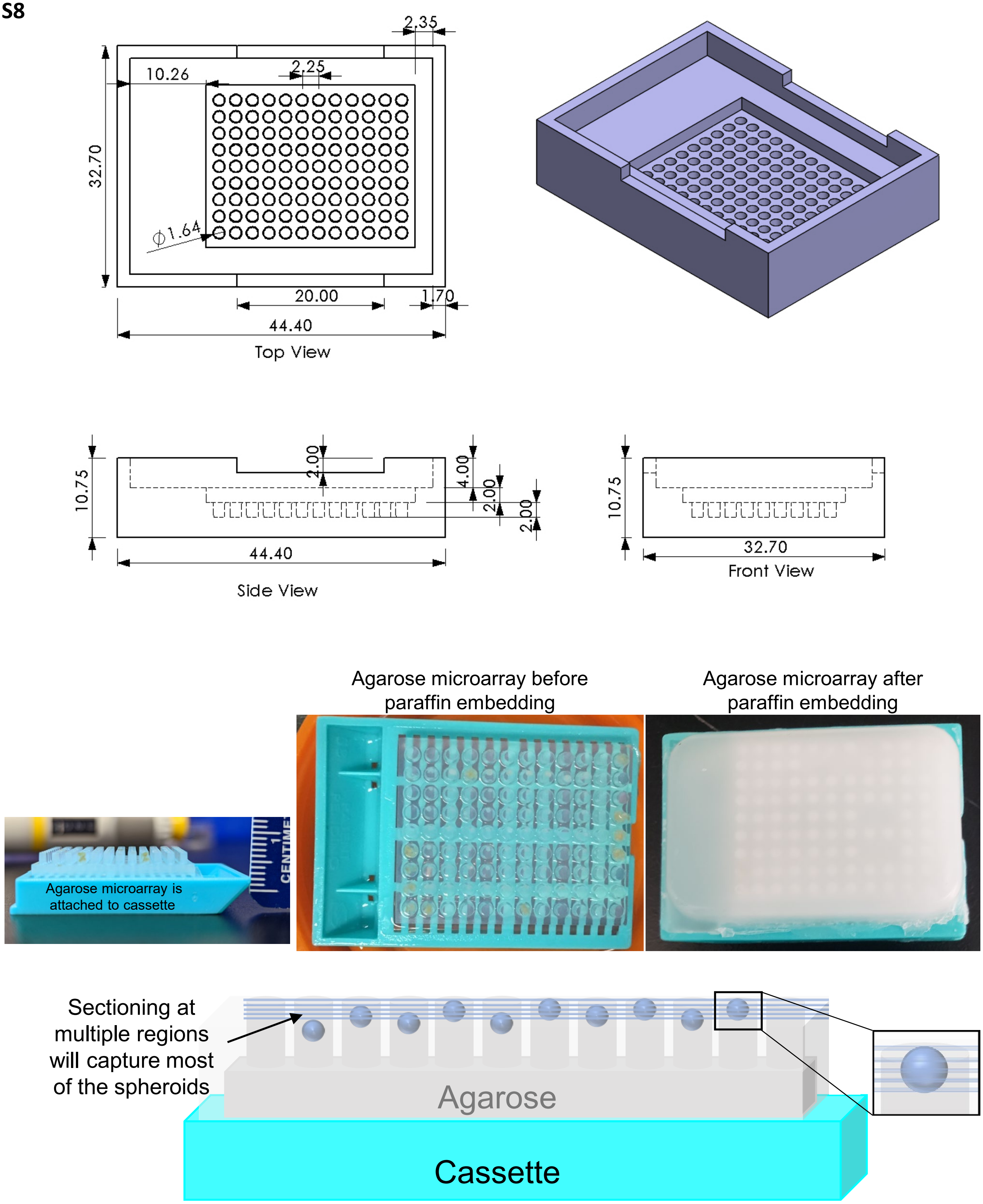
2.35
10.26
2.25
32.70
ϕ1.64
20.00
1.70
44.40
Top View
10.75
2.00
4.00
2.00
2.00
44.40
Side View
10.75
32.70
Front View
Agarose microarray before paraffin embedding
Agarose microarray after paraffin embedding
Agarose microarray is attached to cassette
Sectioning at multiple regions will capture most of the spheroids
Agarose
Cassette

Figure S8: Schematic design of the 3D printed spheroid microarray mold, agarose embedding of spheroids and sectioning diagram.

SVid 1: MC38 cells alone spheroid formation from initial seeding to day 3 of culture acquired by time-lapse imaging.

SVid 2: MC38 + SIS ECM MatriSphere formation from initial seeding to day 3 of culture acquired by time-lapse imaging.

SVid 3: MC38 + Type I Collagen MatriSphere formation from initial seeding to day 3 of culture acquired by time-lapse imaging.

SVid 4: MC38 + Matrigel MatriSphere formation from initial seeding to day 3 of culture acquired by time-lapse imaging.

SVid 5: CT26 cells alone spheroid formation from initial seeding to day 3 of culture acquired by time-lapse imaging.

SVid 6: CT26 + SIS ECM MatriSphere formation from initial seeding to day 3 of culture acquired by time-lapse imaging.

SVid 7: CT26 + Type I Collagen MatriSphere formation from initial seeding to day 3 of culture acquired by time-lapse imaging.

SVid 8: CT26 + Matrigel MatriSphere formation from initial seeding to day 3 of culture acquired by time-lapse imaging.

SVid 9: HT-29 cells alone spheroid formation from initial seeding to day 3 of culture acquired by time-lapse imaging.

SVid 10: HT-29 + SIS ECM MatriSphere formation from initial seeding to day 3 of culture acquired by time-lapse imaging.

SVid 11: HT-29 + Type I Collagen MatriSphere formation from initial seeding to day 3 of culture acquired by time-lapse imaging.

SVid 12: HT-29 + Matrigel MatriSphere formation from initial seeding to day 3 of culture acquired by time-lapse imaging.
